# SLFN11 inhibits hepatocellular carcinoma tumorigenesis and metastasis by targeting RPS4X via mTOR pathway

**DOI:** 10.7150/thno.42869

**Published:** 2020-03-25

**Authors:** Chenhao Zhou, Chunxiao Liu, Wenjie Liu, Wanyong Chen, Yirui Yin, Chia-Wei Li, Jennifer L. Hsu, Jialei Sun, Qiang Zhou, Hui Li, Bo Hu, Peiyao Fu, Manar Atyah, Qianni Ma, Yang Xu, Qiongzhu Dong, Mien-Chie Hung, Ning Ren

**Affiliations:** 1 Department of Liver Surgery, Liver Cancer Institute, Zhongshan Hospital, and Key Laboratory of Carcinogenesis and Cancer Invasion (Ministry of Education), Fudan University, Shanghai, China.; 2 Department of Molecular and Cellular Oncology, The University of Texas MD Anderson Cancer Center, Houston, TX, USA.; 3 Biomedical Research Centre, Zhongshan Hospital, Fudan University, Shanghai, China.; 4 Institute of Fudan Minhang Academic Health System, and Key Laboratory of Whole-period Monitoring and Precise Intervention of Digestive Cancer (SMHC), Minhang Hospital & AHS, Fudan University, Shanghai, China.; 5 Institutes of Biomedical Sciences, Fudan University, Shanghai, China.; 6 Graduate Institute of Biomedical Sciences and Center for Molecular Medicine, China Medical University, Taichung, Taiwan

**Keywords:** hepatocellular carcinoma, Schlafen family member 11, mTOR inhibitor, ribosomal protein S4 X-linked, prognostic biomarker

## Abstract

Hepatocellular carcinoma (HCC) remains one of the most refractory malignancies worldwide. Schlafen family member 11 (SLFN11) has been reported to play an important role in inhibiting the production of human immunodeficiency virus 1 (HIV-1). However, whether SLFN11 also inhibits hepatitis B virus (HBV), and affects HBV-induced HCC remain to be systematically investigated.

**Methods:** qRT-PCR, western blot and immunohistochemical (IHC) staining were conducted to investigate the potential role and prognostic value of SLFN11 in HCC. Then SLFN11 was stably overexpressed or knocked down in HCC cell lines. To further explore the potential biological function of SLFN11 in HCC, cell counting kit-8 (CCK-8) assays, colony formation assays, wound healing assays and transwell cell migration and invasion assays were performed *in vitro*. Meanwhile, HCC subcutaneous xenograft tumor models were established for *in vivo* assays. Subsequently, immunoprecipitation (IP) and liquid chromatography coupled with tandem mass spectrometry (LC-MS/MS) analyses were applied to understand the molecular mechanisms of SLFN11 in HCC. Co-IP, immunofluorescence and IHC staining were used to analyze the relationship between ribosomal protein S4 X-linked (RPS4X) and SLFN11. Finally, the therapeutic potential of SLFN11 with mTOR pathway inhibitor INK128 on inhibiting HCC growth and metastasis was evaluated *in vitro* and *in vivo* orthotopic xenograft mouse models.

**Results:** We demonstrate that SLFN11 expression is decreased in HCC, which is associated with shorter overall survival and higher recurrence rates in patients. In addition, we show that low SLFN11 expression is associated with aggressive clinicopathologic characteristics. Moreover, overexpression of SLFN11 inhibits HCC cell proliferation, migration, and invasion, facilitates apoptosis *in vitro,* and impedes HCC growth and metastasis *in vivo,* all of which are attenuated by SLFN11 knockdown. Mechanistically, SLFN11 physically associates with RPS4X and blocks the mTOR signaling pathway. In orthotopic mouse models, overexpression of SLFN11 or inhibition of mTOR pathway inhibitor by INK128 reverses HCC progression and metastasis.

**Conclusions:** SLFN11 may serve as a powerful prognostic biomarker and putative tumor suppressor by suppressing the mTOR signaling pathway via RPS4X in HCC. Our study may therefore offer a novel therapeutic strategy for treating HCC patients with the mTOR pathway inhibitor INK128.

## Introduction

Hepatocellular carcinoma (HCC) remains one of the most aggressive solid malignancies throughout the world 1, 2, and non-alcoholic fatty liver disease, alcoholic liver disease, and hepatitis B and C viral infections are the major risk factors that drive HCC 3. Although significant advances in hepatectomy, radiofrequency ablation, and liver transplantation have been made in the past decades, the prognosis of HCC patients is still dismal primarily due to the high recurrence and rate of metastasis as well as the disease's tolerance to chemotherapy and targeted therapy 4-6. Therefore, identifying the critical molecular changes and mechanisms involved in HCC progression is urgently needed to develop new therapies for this fatal malignancy.

The Schlafen (SLFN) family proteins have critical roles in the regulation of mammalian biological functions, such as the inhibition of viral replication and induction of immune response 7-9. SLFN11, a member of the human Schlafen family, was previously shown to inhibit the production of human immunodeficiency virus 1 (HIV-1) in a codon- usage-based manner 10. Apart from its antiviral properties, recent studies have indicated that SLFN11 could enhance the sensitivity of cancer cells to DNA-damaging agents (DDAs) and may be a potential robust biomarker to predict response to DDAs in ovarian, lung, and colorectal cancers 11-13. SLFN11 can also block stressed replication forks to promote cell death in response to DNA damage 14. More recently, SLFN11 was reported to have a connection with immune regulation in that it is associated with T-cell infiltration and activation in breast cancer 15.

In the current study, we sought to further our understanding of the functions and underlying mechanisms of SLFN11 in tumorigenesis and metastasis. To this end, we compared the expression of SLFN11 in paired tumor and nontumor liver tissues from HCC patients and analyzed the correlation between SLFN11 expression and survival rates. We also examined the effects of SLFN11 overexpression using *in vitro* assays and animal models. We further showed that SLFN11 interacts with and suppresses oncogenic ribosomal protein S4 X-Linked (RPS4X) which in turn blocks the mTOR signaling pathway. Moreover, inhibition of the mTOR signaling pathway by INK128 or upregulation of SLFN11 expression attenuates HCC tumorigenesis and metastasis. These results together suggest a role of SLFN11 as a prognostic biomarker and a potential therapeutic target for HCC.

## Methods

### Patients and specimens

We obtained 182 liver tumor samples and 182 paired nontumor liver samples from patients who underwent curative hepatectomy in the Department of Liver Surgery, Liver Cancer Institute, Zhongshan Hospital, Fudan University, Shanghai, China, between January 1, 2009 and January 1, 2010 (Fudan LCI cohort 1). We randomly selected 116 paired frozen samples from the Fudan LCI cohort 1 to detect mRNA expression of SLFN11, and 12 paired samples to detect protein expression of SLFN11. The 182 archived paraffin-embedded tissues from Fudan LCI cohort 1 were collected to establish the tissue microarray (TMA). In addition, another independent cohort (Fudan LCI cohort 2) which contains 110 paired HCC samples from patients who underwent hepatectomy at Zhongshan Hospital in 2012 were enrolled in TMA construction as validation cohort.

The enrollment criteria, clinicopathological data collection, and postoperative surveillance were according to our previous study 16. Overall survival (OS) was calculated as the time interval between the date of hepatectomy and death or last follow-up. Recurrence-free survival (RFS) was determined from the date of hepatectomy to tumor recurrence or last follow-up. Written informed consent was obtained from all patients involved in our study, and our study was approved by the research ethics committee of Zhongshan Hospital, Fudan University.

### Cell lines

The normal hepatocyte cell line (L-02) and HCC cell lines Hep3B, SMMC-7721, and PLC/PRF/5 were purchased from the cell bank of Chinese Academy of Sciences (Shanghai, China). HCCLM3 was established at the Liver Cancer Institute, Zhongshan Hospital, Fudan University 17. Cells were cultured in high-glucose Dulbecco's modified Eagle's medium (DMEM) (Gibco, Grand Island, NY, USA) supplemented with 10% fetal bovine serum (Gibco) and 1% penicillin/streptomycin (Gibco) in a humidified 5% CO2 and 37 °C incubator.

### Cell transfection

The lentiviral-based small hairpin RNA (shRNA) targeting SLFN11 or RPS4X and SLFN11 overexpression lentiviruses were constructed by Hanyin Biotechnology Co., Ltd., Shanghai, China. They also provided the control lentivirus with shRNA (Control) and plasmid (Vector). The target sequences of sh1- SLFN11 were 5'-CAGTCTTTGAGAGAGCTTATT-3', sh2-SLFN11 was 5'-GCTCAGAATTTCCGTACTGAA-3', and shRPS4X was 5'- TGACAAGACGGGAGAGAAT-3'. For more details, please see Supplementary Methods.

### RNA extraction and quantitative reverse transcription-polymerase chain reaction (qRT-PCR)

The primers designed in our study were as follows: SLFN11, forward: 5'-CCTGGTTGTGGAACCATCTT-3', and reverse: 5'-CTCTCCTTCTCTTGGTCTCTCT-3'; GAPDH, forward: 5'-CTGGGCTACACTGAGCACC-3', and reverse: 5'-AAGTGGTCGTTGAGGGCAATG-3'; RPS4X, forward: 5'-AGATTTGCATGCAGCGGTTC-3', and reverse: 5'-GGCCTCCTCAGGTGTAATACG-3'. The results were normalized to GAPDH for measuring the relative mRNA expression. Triplicate experiments were performed in each sample. For more details, please see Supplementary Methods.

### Western blot analysis

Total proteins of frozen tissues and cells were extracted by using RIPA buffer (150 mM NaCl, 50 mM Tris [pH7.5], 0.1% sodium dodecyl sulfate (SDS), 1% Triton X-100, 0.5% sodium deoxycholate) supplemented with 1% protease inhibitor cocktail and phosphatase inhibitor cocktail (Bimake, Houston, TX, USA). For drug treatment assays, we treated cells with INK128 (200 nM; SelleckChem, Shanghai, China) for 48 h. Then we extracted the proteins from the cells. For more details, please see Supplementary Methods.

### Antibodies and reagents

The following antibodies were used in our study: anti-SLFN11 (Atlas antibodies, HPA023030), anti-GAPDH (Sigma-Aldrich), anti-RPS4X (Abcam), anti-phospho-RPS6 or anti-phospho-S6 (Ser235/236; Cell Signaling Technology), anti-RPS6 (or S6; Cell Signaling Technology), anti-phospho-eIF4E (Ser209; Cell Signaling Technology), anti-eIF4E (Cell Signaling Technology), anti-Bax (Cell Signaling Technology), anti-Bak (Cell Signaling Technology), anti-Bcl-2 (Cell Signaling Technology), anti-Ki-67 (Abcam), anti-Flag (Cell Signaling Technology), Normal rabbit IgG (Cell Signaling Technology), Alexa Fluor 488 goat anti-mouse IgG (Invitrogen), Alexa Fluor 594 goat anti-rabbit IgG (Invitrogen), anti-SLFN11 (Santa Cruz, sc-374339), and BrdU Cell Proliferation Assay Kit (Cell Signaling Technology). Cisplatin was purchased from MedChem Express. INK128 (also known as sapanisertib, MLN0128, and TAK-228) was purchased from SelleckChem.

### Immunohistochemical (IHC) staining

The construction of HCC patients' TMA was described previously 18. We collected tumor samples from xenograft mouse models. The process of IHC staining has been previously described 16. Briefly, the corresponding specific primary antibodies were applied to stain each tissue sample. Subsequently, the indicated HRP-conjugated secondary antibody was used in incubating the slice at 37 °C for 45 min. We then used diaminobenzidine (DAB) solution (Dako, Denmark) to stain the slice, and we counterstained the nuclei with Harris' hematoxylin. Two experienced pathologists, who had no prior knowledge of patient clinical data, independently assessed the IHC staining. The H-score method was applied for calculating the staining score of each sample, which was to multiply the immunoreaction intensity (negative: 0, weak: 1, moderate: 2, strong: 3) by the staining extent score (0%-100%). According to the H-score, the stained samples were divided into four groups: negative (-; 0), weak (+; 0~1), moderate (++; 1~1.5), and strong (+++; 1.5~3). Samples with a negative or weak H-score were determined to be the low protein expression group, whereas those with a moderate or strong H-score were classified as the high protein expression group.

### Immunofluorescence assay

For SLFN11 and RPS4X staining, cells were fixed in 4% paraformaldehyde at room temperature for 25 min, permeabilized for 20 min in 0.5% Triton X-100, blocked in 5% BSA for 1 h, and then incubated with primary antibodies overnight. After the cells were washed in PBST three times, the indicated secondary antibodies were used to incubate the cells for 1 h at room temperature. Subsequently, 4, 6-diamidino-2- phenylindole (DAPI; Invitrogen) was applied to counterstain the nuclei. After the assay was completed, we used a confocal laser scanning microscope (Olympus Corporation, Tokyo, Japan) to visualize the staining results.

### Cell apoptosis and BrdU incorporation assays

For apoptosis analyses, cells were washed briefly in cold PBS two times and were then resuspended at a concentration of 10^6^ cells/ml in 1X Binding Buffer (BD Biosciences, Franklin Lakes, NJ, USA). 100 µl of the solution was transferred to a 5-ml culture tube followed by the addition of 5 µl of 7-AAD and 5 µl of APC/PE Annexin V solution (BD Biosciences) to every sample. After the cells were incubated in the dark at room temperature for 15 min, they were analyzed by flow cytometry (FACSCalibur, BD Biosciences) within 1 h.

For 5-bromo-2'-deoxyuridine (BrdU) incorporation assays, BrdU cell proliferation assay kit was used to detect the Brdu which was incorporated into cellular DNA. The detailed procedures were conducted according to the manufacturer's instructions.

### Cell proliferation assays

For Cell Counting Kit-8 (CCK-8) assays, HCC cells were seeded into a 96-well plate at 3,000 cells/well with 100 µl of 10% FBS DMEM. According to the protocol of CCK-8 solution (Dojindo, Kumamoto, Japan), 10 µl of CCK-8 solution diluted in 100 µl of complete culture medium replaced the original medium of each group at different time points (24, 48, 72, and 96 h). After the cells were incubated in the dark at 37 °C for an additional 2 h, we detected viable cells by using absorbance at a 450-nm wavelength.

For colony formation assays, we planted HCC cells into the six-well plate at a density of 1,000 cells/well and then cultured them in complete culture medium at 37 °C for 14 d. After the cells were gently washed in PBS twice, they were fixed with 4% paraformaldehyde for 30 min. Then 0.1% (w/v) crystal violet was applied for staining the fixed cells for 30 min. ImageJ software (National Institute of Health, Bethesda, MD, USA) was used to count the numbers of colonies. Three independent assays were performed to analyze cell proliferation abilities.

### Wound healing assays

HCC cells were seeded in a six-well plate. When the cells formed a tight cell monolayer, a 200-µl plastic pipette tip was used to make a scratch. To remove the cell debris, we washed the cells in PBS three times. After we removed the complete DMEM, we added serum-free DMEM into each well. Wound photographs were recorded at the indicated times by an IX71 inverted microscope (Olympus Corporation) and analyzed by ImageJ software. All assays were conducted three times in our study.

### Transwell cell migration and invasion assays

Transwell assays were conducted in 24-well transwell plates (pore size: 8 µm; Corning, NY, USA) to assess the migratory and invasive capacities of HCC cells. For migration assays, we placed 4 × 10^4^ HCC cells in 200 µl of serum-free DMEM in the upper chamber and then added 500 µl of DMEM containing 30% FBS to the lower chamber.

For the invasion assays, we precoated the chamber inserts with 50 µl of 1:6 mixture of Matrigel (BD Biosciences) and DMEM for about 2 h in a 37 °C incubator. Then we seeded 8 × 10^4^ HCC cells in the upper chamber. The lower chamber also had 500 µl of DMEM containing 30% FBS. After the cells were incubated for 48 h, we used 4% paraformaldehyde to fix the cells that had migrated or invaded to the lower surface of the membrane. Then crystal violet was applied for staining the fixed cells for 15 min. Five random 100× microscopic fields were selected to count the stained cells by using an IX71 inverted microscope (Olympus Corporation). We repeated all of our assays three times in our study.

### Cell viability assays

HCC cells were seeded into a 96-well plate at 3,000 cells/well with 100 µl of 10% FBS DMEM. After overnight incubation, the complete medium containing different concentrations of cisplatin (1, 5, 10, 20, 40, 60, 80, 100 μM) replaced the original medium of each group for 72 h. Then the cell viability was determined by CCK-8 assay. The drug half-maximum inhibitory concentration (IC50) was calculated by GraphPad Prism 8 Software (San Diego, CA, USA).

### Co-Immunoprecipitation and mass spectrometry (Co-IP/MS)

Co-IP assay and MS were conducted as previously described 19. Briefly, we extracted the total proteins from HCC cells via IP lysis buffer. The proteins were then quantified by the Pierce™ BCA Protein Assay Kit. The relevant antibodies and protein A/G beads were added into the protein lysates. Afterward, the lysates were incubated at 4 °C overnight. Pre-cold IP lysis buffer was used to wash the immunocomplex samples 5 times. Then the samples were boiled in SDS-PAGE loading buffer and then analyzed by Western blotting.

For MS analysis, the gels were first cut into small pieces, decolorized by certain solution, and dried with 100% acetonitrile. Then the gels were digested, and the peptides were obtained from them. The peptides were detected by liquid chromatography coupled with tandem mass spectrometry (LC-MS/MS) on a Q Exactive (QE) mass spectrometer. Mascot (Matrix Science, London, UK; version 2.3.0), an engine that can search the uniprot_human database, which contains 20205 protein sequences, was used for analyzing the LC-MS/MS data. The Mascot parameters were set as follows: Enzyme = “Trypsin”, fragment mass tolerance = “± 0.05 Da”, mass values = “Monoisotopic”, variable modifications = “Oxidation (M)”, peptide mass tolerance = “10 ppm”, instrument type = “ESI-FTCR”, max missed cleavages = “1”, fixed modifications = “Carbamidomethyl (C), *P* value < 0.05, ion score > 20”. In the end, Ingenuity Pathway Analysis (IPA) software (http://www.ingenuity.com/products/ipa) was applied for analyzing the significant pathways and functions involved in the identified proteins.

### Mouse xenograft study

All male BALB/c nude mice (4-6 weeks of age) were purchased from the Shanghai SLAC Laboratory Animal Co., Ltd., and raised in a standard pathogen-free (SPF) environment in the experimental animal center of Zhongshan Hospital, Fudan University. Our project was conducted under guidelines approved by the Institutional Animal Care and Ethics Committee of Zhongshan Hospital, Fudan University. For more details, please see Supplementary Methods.

### Statistical analysis

SPSS 23.0 software was used for statistical analyses (IBM, Armonk, NY, USA). The continuous values of experiments were recorded as means ± standard deviation (SD). According to the distribution of our experimental data, Pearson Chi-squared test, Fisher's exact test, Mann-Whitney *U* test, or two-tailed Student's *t*-test were used to analyze the statistical *P* values between different groups. The OS and RFS were analyzed by Kaplan-Meier's method (log-rank test). Significant variables related to OS and RFS were recognized by univariate Cox proportional hazards regression analyses (*P* < 0.05). Then multivariate Cox proportional hazards regression analyses were performed in a backward manner to analyze the independent prognostic factors by adopting all significant variables from univariate analyses. The 'survivalROC' package of R software (version 3.6.3) was used to conduct receiver operating characteristic (ROC) analyses. A *P* value of < 0.05 (two-tailed) was considered as statistically significant.

## Results

### SLFN11 is downregulated in human HCC and correlates with poor prognosis

**Figure 1:**
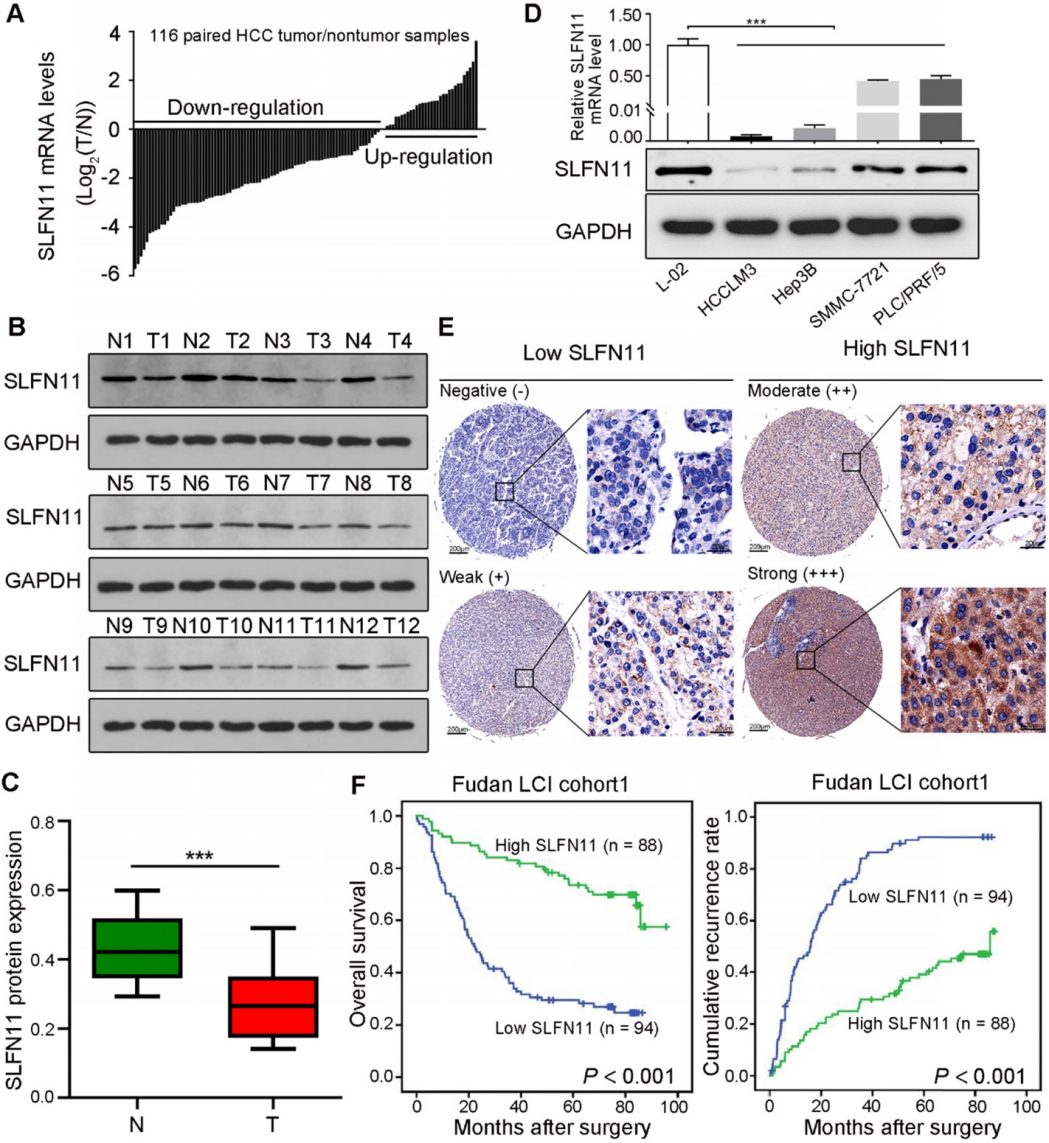
** SLFN11 is downregulated in human HCC and correlates with poor prognosis. (A)** qRT-PCR analysis of SLFN11 mRNA expression in 116-paired tumor and nontumor liver tissues. **(B, C)** Western blot of SLFN11 protein expression in 12-paired nontumor (N) and tumor (T) liver tissues. **(D)** mRNA and protein expression level of SLFN11 in a normal liver cell line (L-02)and four HCC cell lines (HCCLM3, Hep3B, SMMC-7721, and PLC/PRF/5). **(E)** Representative IHC staining images indicating low and high expression of SLFN11 in HCC tissue microarray. Scale bars = 200 μm or 20 μm, respectively. **(F)** Kaplan-Meier curves for overall survival and recurrence-free survival based on SLFN11 expression in the Fudan LCI cohort 1. *** *P* < 0.001.

To investigate the potential role of SLFN11 in HCC, we first examined its mRNA expression in paired tumor and nontumor liver tissues from 116 patients with HCC by qRT-PCR. The results revealed that SLFN11 was downregulated in 72.41% of tumor tissues (84/116) compared with nontumor liver tissues (**Figure 1A**). Western blot analysis indicated that SLFN11 protein expression was also downregulated in 12-paired samples randomly selected from the 116 (**Figure 1B-C**). In a panel of four HCC cell lines (HCCLM3, Hep3B, SMMC-7721, and PLC/PRF/5), both mRNA and protein levels of SLFN11 were relatively lower compared with that in the L-02 normal liver cells (**Figure 1D**).

To further investigate the prognostic value of SLFN11 expression in HCC, IHC analysis was used to assess TMA from patients in the Fudan LCI cohort 1 (N = 182) and Fudan LCI cohort 2 (N = 110) with complete clinicopathologic characteristics and follow-up data. According to the IHC scores, patients were dichotomized into low (-/+) or high (++/+++) SLFN11 expression group (**Figure 1E**). In Fudan LCI cohort 1, the clinicopathological analysis indicated that SLFN11 expression negatively correlated with high levels of serum alpha-fetoprotein (AFP; *P* < 0.001), a marker widely used to detect HCC, large tumor size (*P* = 0.005), presence of microvascular invasion (*P* = 0.025), advanced Barcelona Clinic Liver Cancer (BCLC) stage (*P* = 0.038), and advanced tumor-nodes-metastasis (TNM) stage (*P* = 0.030) (**Table S1**). Likewise, in Fudan LCI cohort 2, SLFN11 expression negatively correlated with high levels of serum AFP (*P* = 0.022), serum CA19-9 (*P* = 0.002), presence of microvascular invasion (*P* = 0.045), advanced BCLC stage (*P* = 0.014), and advanced TNM stage (*P* = 0.033) (**Table S2**).

Survival analysis of the Fudan LCI cohort 1 revealed that patients in low SLFN11 group had noticeably shorter OS and RFS than did those in high SLFN11 group (*P* < 0.001, *P* < 0.001, respectively) (**Figure 1F**). Similar results were acquired from the other independent HCC patient cohort (Fudan LCI cohort 2) (**Figure S1A-B**). In addition, by adopting all significant variables in univariate analyses, multivariate Cox regression analysis showed that low SLFN11 expression was an independent prognostic factor for OS (Fudan LCI cohort 1: hazard ratio [HR] = 3.142, 95% confidence interval [CI]: 1.957-5.045, *P* < 0.001; Fudan LCI cohort 2: HR = 3.924, 95% CI: 2.169-7.099, *P* < 0.001) and RFS (Fudan LCI cohort 1: HR = 3.659, 95% CI: 2.414-5.547, *P* < 0.001; Fudan LCI cohort 2: HR = 4.977, 95% CI: 2.639-9.388, *P* < 0.001) (**Table S3** and**Table S4**). Furthermore, we reanalyzed the survival curves and multivariate models for OS and RFS by categorizing patients into four groups according to SLFN11 IHC score: negative (-), weak (+), moderate (++), and strong (+++). In both training and validation cohort, patients with lower SLFN11 expression were confirmed to be significantly associated with shorter OS duration and higher recurrence rates (**Figure S1C-F**,**Table S3** and**Table S4**). Moreover, based on the multivariable models containing SLFN11 generated in the training cohort, time-dependent ROC analyses for the OS and RFS prediction signify the accuracy of SLFN11 expression as an adjunct for biomarker analysis in both training and validation cohort (**Figure S2A-D**). Together, these findings indicated that low SLFN11 expression in HCC may manifest as poor prognosis and tumor aggressiveness.

### SLFN11 inhibits cell proliferation, migration, and invasion, and facilitates apoptosis* in vitro*

**Figure 2:**
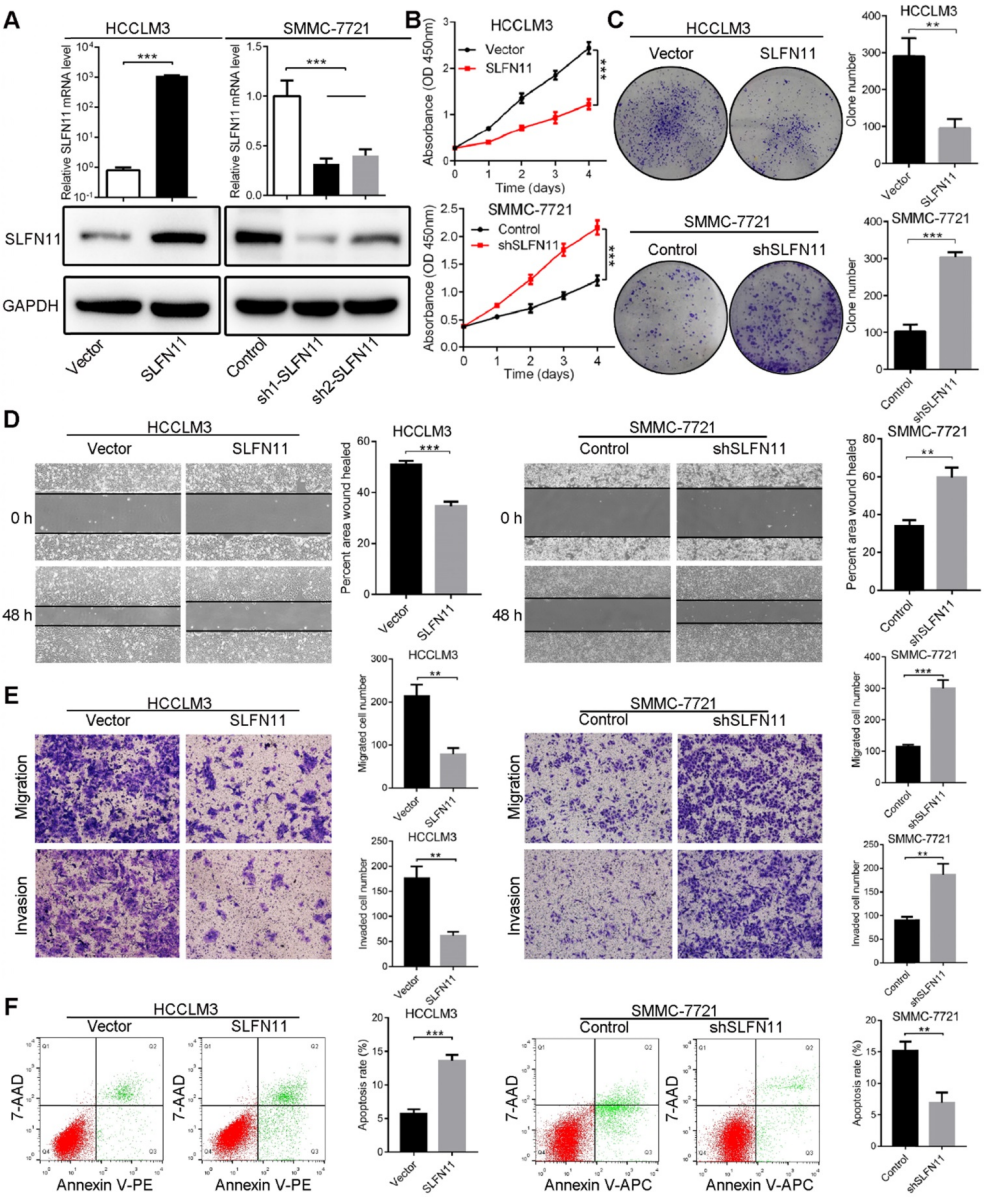
** SLFN11 inhibits cell proliferation, migration, invasion, and facilitates apoptosis *in vitro*. (A)** The overexpressing and knockdown efficiency of SLFN11 was verified by qRT-PCR and Western blot assays in HCCLM3 and SMMC-7721 cells. **(B)** Effects of SLFN11 overexpression and knockdown on cell proliferation by CCK-8 in HCCLM3 and SMMC-7721 cells. **(C)** Effects of SLFN11 overexpression and knockdown on cell proliferation by colony formation assays in HCCLM3 and SMMC-7721 cells. **(D)** Effects of SLFN11 overexpression and knockdown on cell migratory abilities by wound healing assays in HCCLM3 and SMMC-7721 cells. **(E)** Effects of SLFN11 overexpression and knockdown on cell migratory and invasive capacities by transwell assays in HCCLM3 and SMMC-7721 cells. **(F)** Effects of SLFN11 overexpression and knockdown on cell apoptosis by flow cytometry in HCCLM3 and SMMC-7721 cells. ** *P* < 0.01, *** *P* < 0.001.

Based on expression levels of SLFN11 in HCC cell lines, we generated stable SLFN11-overexpressing (SLFN11 OE) cells in low SLFN11-expressing HCCLM3 and Hep3B cell lines, and SLFN11 knockdown (SLFN11 KD; SLFN11 short hairpin RNA) cells in high SLFN11-expressing SMMC-7721 and PLC/PRF/5 cell lines. The overexpression and knockdown efficiency of SLFN11 was verified by comparison with the vector or control at both mRNA and protein levels (**Figure 2A** and **Figure S3A**). Among the SLFN11 shRNAs tested, shRNA-1 demonstrated the best inhibitory effect against *SLFN11* both in SMMC-7721 and PLC/PRF/5 cell lines, and thus was selected for further experiments.

To explore the potential function of SLFN11 in HCC cells, we conducted a series of *in vitro* assays. Results from cell counting and colony formation assays indicated that the proliferation ability of SLFN11 OE HCCLM3 (**Figure 2B-C**) and Hep3B cells (**Figure S3B-C**) was decreased compared with the vector control cells. In contrast, knocking down SLFN11 led to significantly increased proliferation capacity of SMMC-7721 (**Figure 2B-C**) and PLC/PRF/5 cells compared with the control cells (**Figure S3B-C**). Wound healing assays showed that overexpression of SLFN11 in HCCLM3 and Hep3B cells impeded cell migratory abilities, whereas the migratory potentials of SMMC-7721-shSLFN11 and PLC/PRF/5-shSLFN11 cells were noticeably enhanced compared with the corresponding control cells (**Figure 2D**, **Figure S3D**). In addition, results from transwell assays indicated that the migratory and invasive capacities were greatly hindered by overexpression of SLFN11 in HCCLM3 and Hep3B cells, whereas SLFN11 KD HCC cells had significantly higher cell migratory and invasive capacities than did control cells (**Figure 2E**,**Figure S3E**). Moreover, in SLFN11 OE HCCLM3 cells, the apoptosis rate, as determined by flow cytometry, was 13.62% ± 0.87% compared with a rate of 5.71% ± 0.64% in vector control cells; the cell apoptosis rate of SLFN11 KD SMMC-7721 cells was 6.89% ± 1.65% compared with a rate of 15.18% ± 1.44% in shRNA control cells (**Figure 2F**). Similar results were also observed in Hep3B and PLC/PRF/5 cells after SLFN11 overexpression or knockdown (**Figure S3F**). Additionally, to test whether S phase of cell cycle was affected by the expression of SLFN11, the BrdU incorporation assays were performed. **Figure S4** showed that overexpression of SLFN11 in HCCLM3 decreased cellular proliferation (*P* < 0.001), whereas SLFN11 KD SMMC-7721 cells exhibited higher cell proliferation rate (*P* < 0.001). The results indicated that SLFN11 might hinder tumor cells into S phase of cell cycle or the progression of S phase. Together, these data revealed that SLFN11 inhibits cell proliferation, migration, and invasion, and induces cell apoptosis.

### SLFN11 attenuates HCC progression *in vivo*

**Figure 3:**
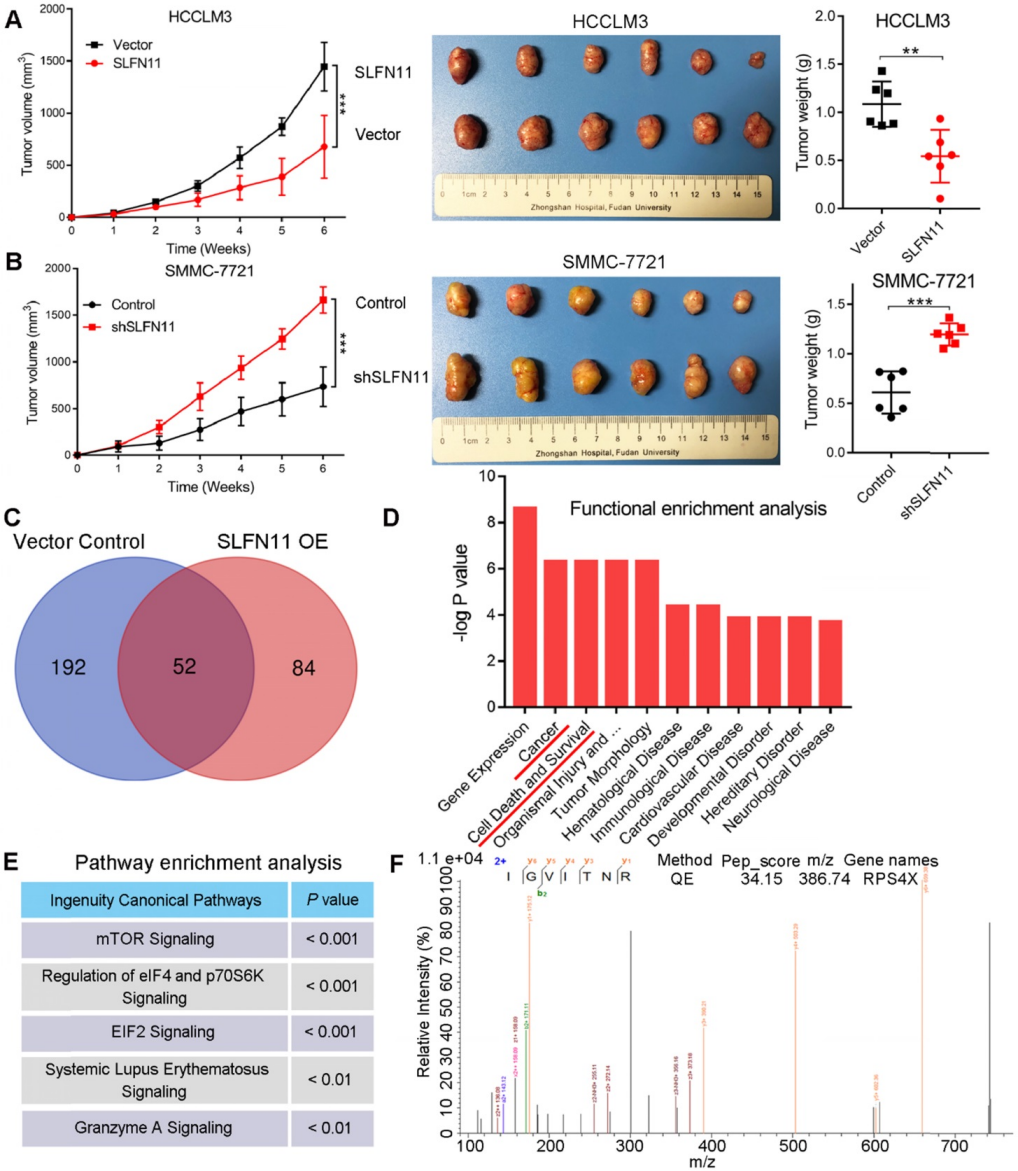
** SLFN11 inhibits HCC progression *in vivo*, as well as the proteomic analyses of SLFN11 complexes by liquid chromatography coupled with tandem mass spectrometry (LC-MS/MS). (A)** Effects of SLFN11 overexpression on HCC progression by establishment of subcutaneous xenograft mouse models. Tumor growth curves and weight of xenografts derived from HCCLM3-Vector or HCCLM3-SLFN11 cells are shown. **(B)** Effects of SLFN11 knockdown on HCC progression by establishment of subcutaneous xenograft mouse models. Tumor growth curves and weight of xenografts derived from SMMC-7721-Control or SMMC-7721-shSLFN11 cells are shown. **(C)** Venn diagram shows the overlapping and unique proteins identified from the complexes related to SLFN11 overexpression and the vector control cells. **(D)** Functional enrichment analysis of the unique proteins related to SLFN11 with ingenuity pathway analysis (IPA) software. **(E)** Pathway enrichment analysis of the unique proteins related to SLFN11 with IPA software. **(F)** The peptide spectrum of RPS4X by LC-MS/MS assay. The N-terminal and C-terminal collision-induced dissociation fragment ions are indicated by b and y, respectively. ** *P* < 0.01, *** *P* < 0.001. Abbreviations: QE, Q Exactive.

To further validate our above results *in vitro*, we established HCC xenograft tumor models. Nude mice were subcutaneously injected with HCCLM3- SLFN11, SMMC-7721-shSLFN11, or their associated control cells, and tumor growth was monitored for 6 weeks at which. As shown in the growth curves, tumors in the HCCLM3-vector and SMMC-7721- shSLFN11 groups grew faster than did those in the HCCLM3-SLFN11 and SMMC-7721-control groups over the same time period, respectively (**Figure 3A-B, left**). Tumors derived from the HCCLM3-vector and SMMC-7721-shSLFN11 groups were significantly larger (*P* < 0.001, *P* < 0.001, respectively; **Figure 3A-B, middle**] and weighed more (*P* = 0.004, *P* < 0.001, respectively; **Figure 3A-B, right**) than those derived from the HCCLM3-SLFN11 and SMMC-7721-control groups. Hence, our results suggested that SLFN11 plays a critical role in inhibiting HCC tumorigenesis and progression *in vivo*.

### SLFN11 physically associates with RPS4X and blocks the mTOR signaling pathway

To understand the molecular mechanisms of SLFN11 in HCC progression, we examined potential SLFN11 interacting proteins in stable SLFN11 OE and vector control HCCLM3 cells by immunoprecipitation (IP) and liquid chromatography coupled with tandem mass spectrometry (LC-MS/MS) analyses. SLFN11- associated protein complexes from HCCLM3 cells were isolated by using paramagnetic beads coated with anti-Flag mAbs. Affinity purification and MS analyses were carried out using the protein complexes. Proteomic analysis identified 84 and 192 proteins unique to the complexes related to SLFN11 in the SLFN11 OE and vector control group, respectively, whereas 52 were common between both groups (**Figure 3C**). The list of 84 unique proteins that interact with SLFN11 was shown in **Table S5**. Functional enrichment analysis further indicated that cancer and cell death and survival were ranked as the top 2 and 3 molecular networks, respectively, in the cluster of SLFN11-enriched proteins (**Figure 3D**). Pathway enrichment analysis of these proteins revealed the top three affected canonical signaling pathways: mTOR (*P* < 0.001), eIF4/p70S6K (*P* < 0.001), and EIF2 signaling (*P* < 0.001) (**Figure 3E**). Both eIF4/p70S6K and EIF2 are downstream of the mTOR signaling pathway 20, 21. We repeatedly identified the oncogenic ribosomal protein S4 X-linked (RPS4X) as an SLFN11-interacting protein in the mTOR, eIF4/ p70S6K, and EIF2 signaling pathways (**Figure 3F**).

**Figure 4:**
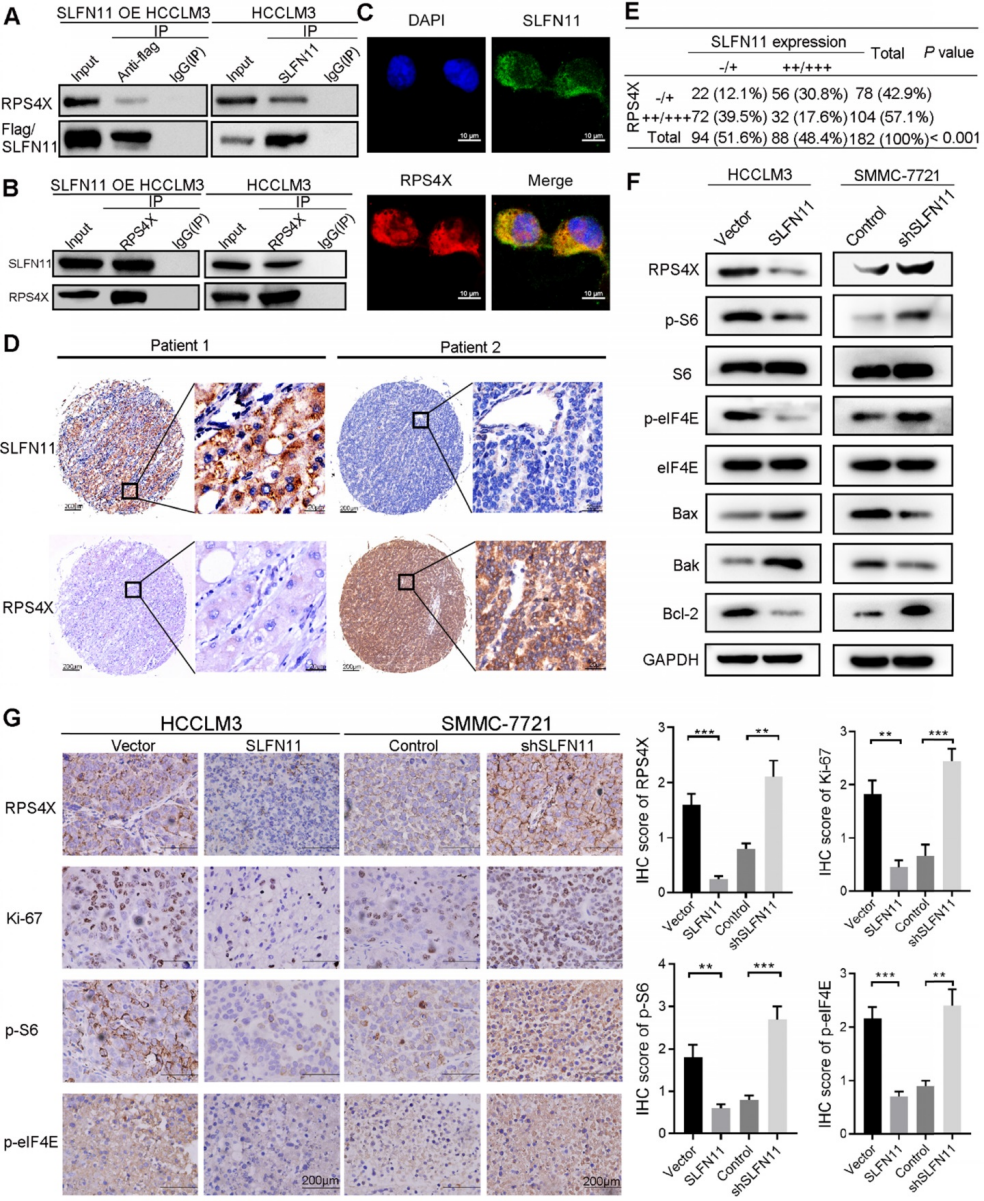
** SLFN11 blocks the mTOR signaling pathway and physically associates with RPS4X. (A, B)** Co-immunoprecipitation (Co-IP) assays were conducted in HCCLM3 cells transfected with a vector containing flag-tagged SLFN11 and HCCLM3 cells; IgG was used as control. **(C)** Confocal microscopy scan of immunofluorescence staining shows that SLFN11 (green) co-localized with RPS4X (red) in the HCCLM3 cells. DAPI was used for nuclear staining. Scale bars = 10 μm. **(D)** Representative IHC staining of HCC tumors for SLFN11 and RPS4X expression. Scale bars = 200 μm or 20 μm, respectively. **(E)** Correlations between SLFN11 and RPS4X expression levels in HCC patients in the Fudan LCI cohort 1. *P* value was calculated by Pearson Chi-squared test; -/+, negative or weak expression; ++/+++, moderate or strong expression. **(F)** Changes in the mTOR signaling pathway and apoptotic-related proteins were detected by Western blotting for SLFN11-OE in HCCLM3 cells and SLFN11 knockdown in SMMC-7721 cells.** (G)** Representative IHC images of xenograft tumors from nude mice subcutaneously injected with corresponding transfected HCCLM3 and SMMC-7721 cells stained with RPS4X, Ki-67, p-S6 and p-eIF4E. Scale bars = 200 μm. Histograms (right) show the IHC score ± SD in each group. ** *P* < 0.01, *** *P* < 0.001.

Next, we validated the relationship between RPS4X and SLFN11 by co-immunoprecipitation (Co-IP) and immunofluorescence staining. Endogenous RPS4X was co-immunoprecipitated by Flag and SLFN11 antibodies whereas the endogenous SLFN11 and Flag-tagged SLFN11 were reciprocally co-immunoprecipitated by the RPS4X antibody in SLFN11 OE HCCLM3 and HCCLM3 cells (**Figure 4A-B**). Results from immunofluorescence staining indicated that SLFN11 and RPS4X co-localized in HCCLM3 cells (**Figure 4C**).

To determine whether a clinical correlation exists between SLFN11 and RPS4X expression, we analyzed their expression in 182 HCC tissues from the Fudan LCI cohort 1 by IHC staining (**Figure 4D**; representative images shown). As shown in Figure 4E, SLFN11 expression correlated negatively with RPS4X (*P* < 0.001). Specifically, about 77% of the tumor tissues (72 of 94) with low SLFN11 expression showed moderate or strong RPS4X staining and 64% (56 of 88) of those with high SLFN11 expression displayed negative or weak RPS4X staining (**Figure 4E**).

The translational initiation factor eIF4E has been shown to promote the recruitment of the 40S ribosomal subunit 22, which contains both RPS4X and S6 ribosomal proteins 23, 24. Moreover, both S6 and eIF4E regulate the mTOR signaling pathway. Hence, we also examined the effects of SLFN11 expression on S6, eIF4E, and their phosphorylation. The results showed that SLFN11 overexpression downregulated phosphorylation of S6 and eIF4E, which is indicative of inhibition of the mTOR signaling pathway (**Figure 4F, left**). In contrast, knockdown of SLFN11 enhanced the activity of the mTOR signaling pathway (**Figure 4F, right**). We also examined the expression levels of cell death and survival-related proapoptotic proteins Bax and Bak in SLFN11 OE HCCLM3 cells and SLFN11 KD SMMC-7721 cells. Both Bax and Bak increased with overexpression of SLFN11 and decreased with knockdown of SLFN11 whereas opposing effects were observed for antiapoptotic protein Bcl-2 (**Figure 4F**). Moreover, IHC staining of the xenograft tumors from previous data indicated that RPS4X, p-S6, p-eIF4E, and cell proliferation marker Ki-67 were markedly suppressed in SLFN11 OE tumors whereas expression of these markers was substantially enhanced in shSLFN11 tumors (**Figure 4G**). Collectively, these results suggested that SLFN11 physically interacts with RPS4X and attenuates S6 and eIF4E phosphorylation, leading to inhibition of the mTOR signaling pathway.

### RPS4X is an essential factor in SLFN11-mediated inhibition of mTOR signaling pathway

**Figure 5:**
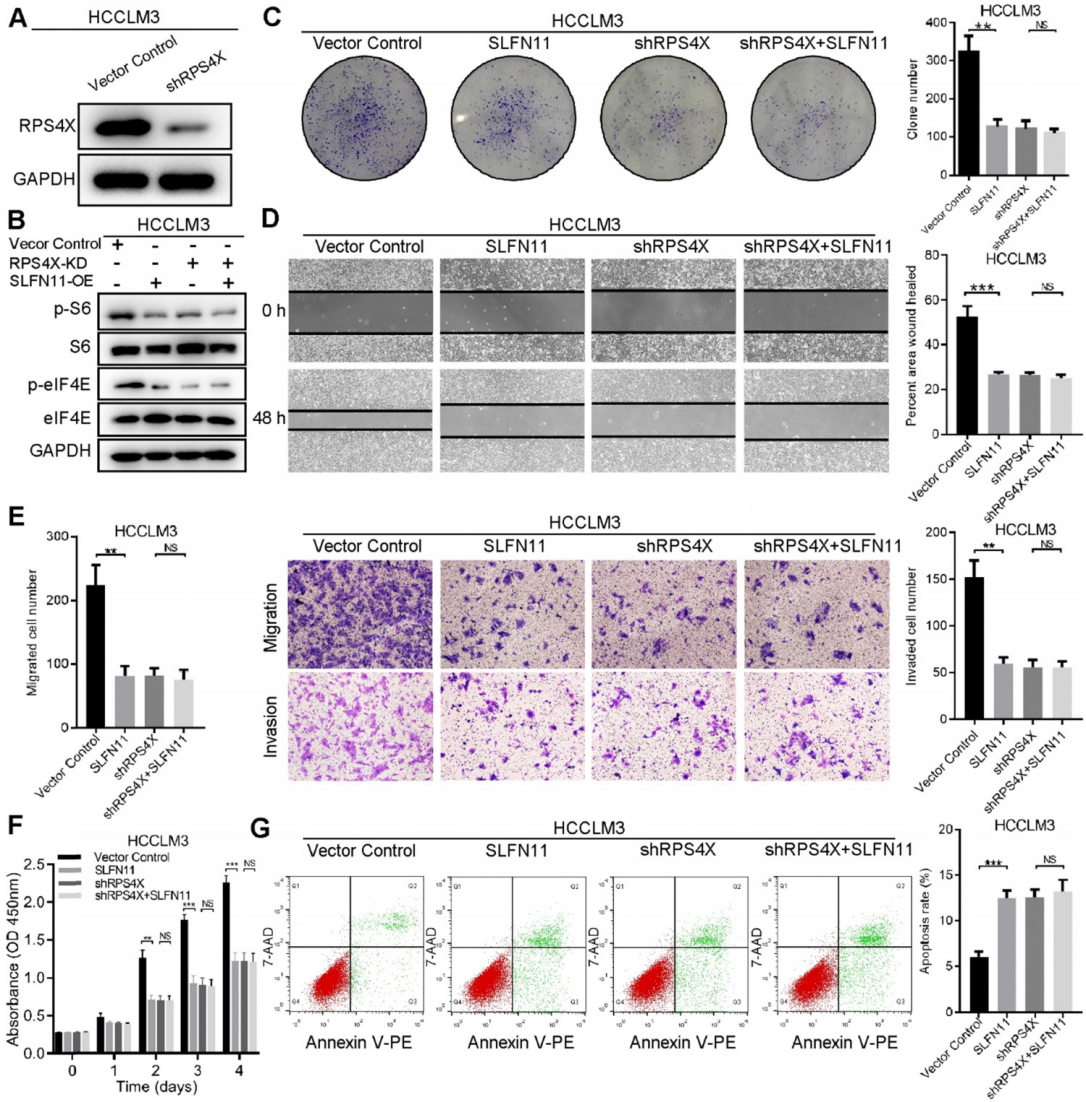
** RPS4X is an essential factor in SLFN11-mediated inhibition of the mTOR signaling pathway. (A)** Western blot of the knockdown efficiency of RPS4X in HCCLM3 cells. **(B)** Western blot indicates that once RPS4X was knocked down in HCCLM3 cells, regardless of whether SLFN11 was overexpressed, the phosphorylation of S6 and eIF4E were inhibited at almost the same level. **(C)** Colony formation assays were conducted to study cell proliferation of HCCLM3-VectorControl cells and HCCLM3-SLFN11 cells with or without RPS4X knockdown. **(D)** Wound healing assays were performed to detect cell migratory abilities of HCCLM3-VectorControl cells and HCCLM3-SLFN11 cells with or without RPS4X knockdown.** (E)** Transwell assays were used to investigate the cell migratory and invasive capacities of HCCLM3-VectorControl cells and HCCLM3-SLFN11 cells with or without RPS4X knockdown. **(F)** CCK-8 assays were conducted to determine the cell proliferation of HCCLM3-VectorControl cells and HCCLM3-SLFN11 cells with or without RPS4X knockdown. **(G)** Cell apoptosis was assessed by flow cytometry in HCCLM3-VectorControl cells and HCCLM3-SLFN11 cells with or without RPS4X knockdown. ** *P* < 0.01, *** *P* < 0.001, NS, not significant.

To determine whether RPS4X plays an essential role in SLFN11-mediated inhibition of HCC tumorigenesis and progression, we knocked down RPS4X expression in HCCLM3 and SMMC-7721 cells by shRNA (**Figure 5A, Figure S5A**). The effects of RPS4X knockdown on S6 and eIF4E phosphorylation, as well as on cellular proliferation, migration and invasion, and apoptosis, were similar to that of SLFN11 overexpression (**Figure 5B-G**); the extent of phosphorylation and biological functions could not be further enhanced in HCCLM3-SLFN11 cells with RPS4X knockdown. In SMMC-7721-shSLFN11 cells, knocking down RPS4X dampened the shSLFN11- mediated increase in S6 and eIF4E phosphorylation, proliferation, and migration and invasion, and restored apoptosis to levels similar to that of control cells (**Figure S5B-G**). On the basis of the above results, we concluded that RPS4X is required for SLFN11-mediated inactivation of the mTOR signaling pathway.

### SLFN11 expression in combination with mTOR inhibitor exhibits therapeutic potential in HCC

**Figure 6:**
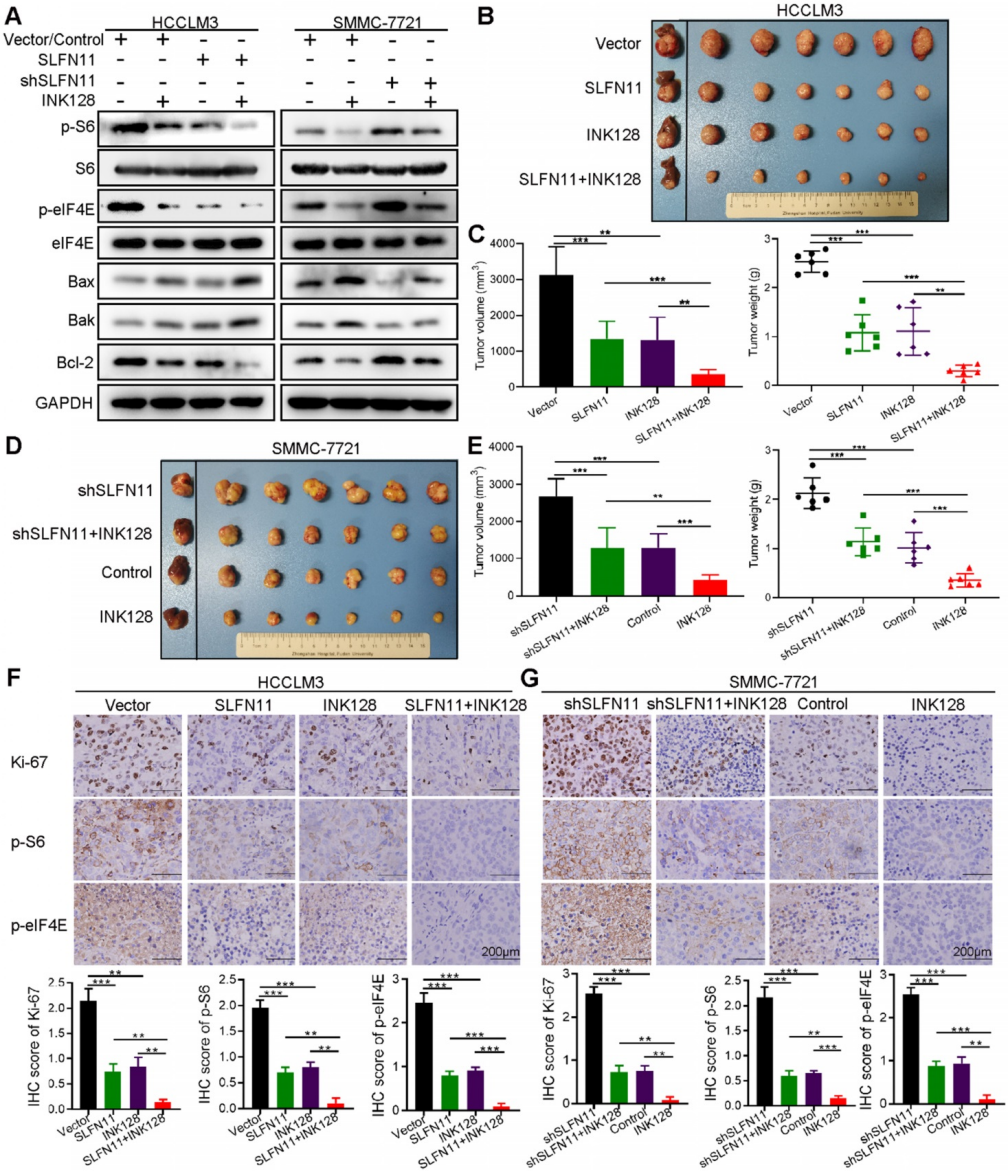
***In vivo* efficacy of combined SLFN11 expression and mTOR inhibition. (A)** Western blots of p-S6, p-eIF4E, and the proteins involved in cell apoptosis in HCCLM3-Vector, HCCLM3-SLFN11, SMMC-7721-Control, and SMMC-7721-shSLFN11 cells that had been treated with or without INK128 (200 nM). **(B, C)** Tumor volume and weight of orthotopic xenograft models derived from HCCLM3-Vector and HCCLM3-SLFN11 cells treated as indicated. **(D, E)** Tumor volume and weight of orthotopic xenograft models derived from SMMC-7721-Control and SMMC-7721-shSLFN11 cells treated as indicated. **(F, G)** Representative IHC images of orthotopic nude mouse tumor tissues for expression of Ki-67, p-S6, and p-eIF4E from HCCLM3-Vector, HCCLM3-SLFN11, SMMC-7721-Control, and SMMC-7721-shSLFN11 cells treated as indicated. Scale bars = 200 μm. Histograms (bottom) show the IHC score ± SD in each group. ** *P* < 0.01, *** *P* < 0.001.

SLFN11 has been reported to associate with tumor cell sensitivity to several clinical drugs, such as irinotecan and cisplatin 25, 26. The cell viability assays of our study validated that SLFN11 may enhance HCC cell sensitivity to cisplatin (**Figure S6**). Thus, we next investigated the combination of SLFN11 expression with mTOR inhibitor INK128 (also known as sapanisertib, MLN0128, or TAK-228). First, we examined the expression levels of representative markers in the mTOR signaling pathway and those associated with cell apoptosis in HCC cells by Western blot analysis. As shown in Figure 6A left, p-S6, p-eIF4E and Bcl-2 indicated a decrease while Bax and Bak showed an increase when we overexpressed SLFN11 in HCCLM3 cells or treated them with INK128. Treatment of SMMC-7721- shSLFN11 cells with INK128 inhibited the mTOR signaling pathway and increased cell apoptosis (**Figure 6A**). The combination of high SLFN11 expression and INK128 treatment led to a substantial decrease in the protein levels involved in the mTOR signaling pathway than that with either intervention alone (**Figure 6A**).

**Figure 7:**
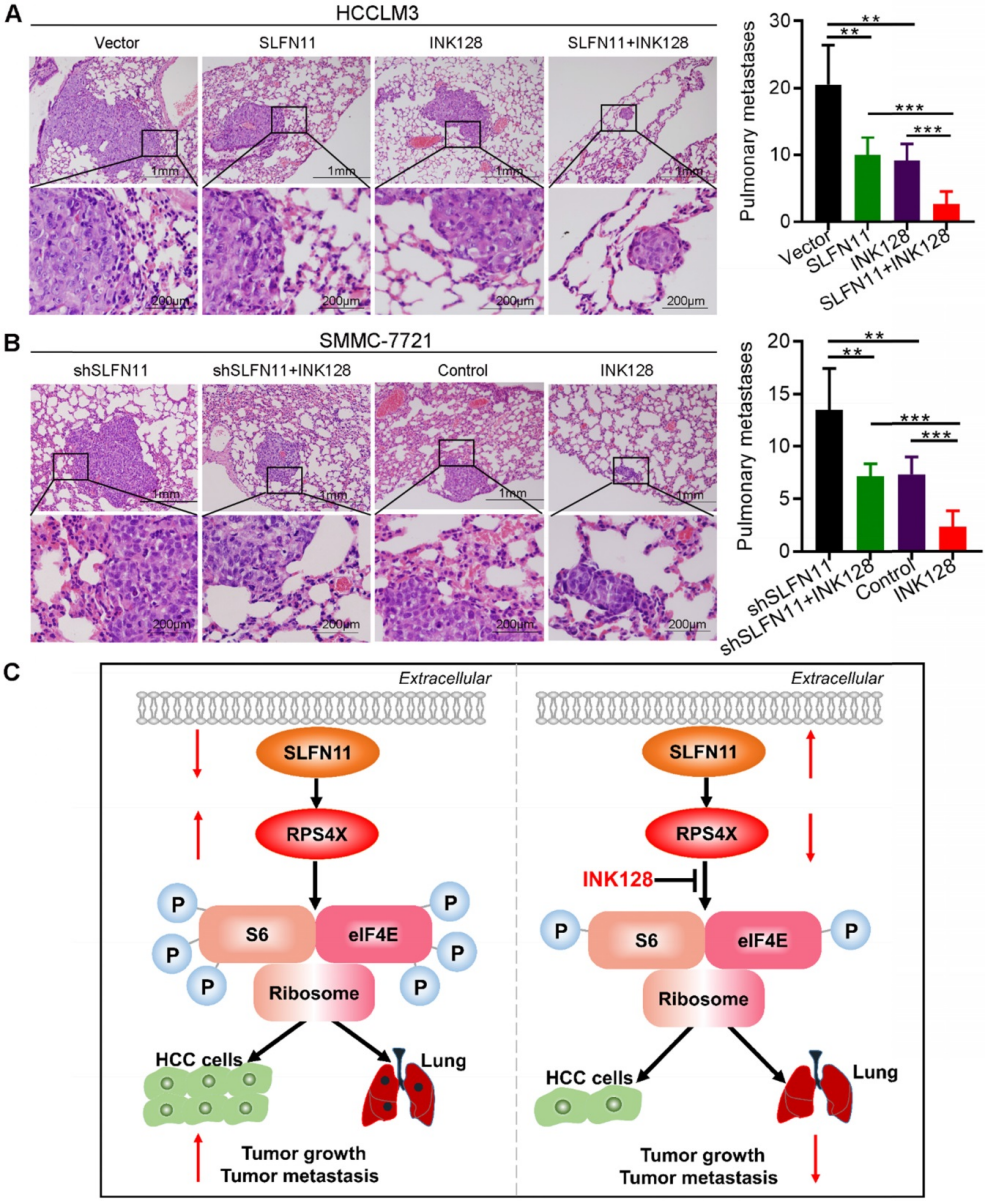
** SLFN11 inhibits tumor metastasis *in vivo*. (A, B)** Left panel: Representative hematoxylin and eosin (HE) staining images indicate the effects of SLFN11 and INK128 on lung metastasis from orthotopic nude mice in HCCLM3 and SMMC-7721 cells. Scale bars = 1 mm and 200 μm. Right panel: Histograms show metastatic nodules in the lungs with a mean ± SD in each group. ** *P* < 0.01, *** *P* < 0.001. **(C)** A schematic illustrating the role of SLFN11 in regulating HCC tumorigenesis and metastasis.

To further evaluate the effects of SLFN11 and INK128 on tumor growth and metastasis, we established orthotopic xenograft mouse models of HCC (6 mice per group). The size and weight of SLFN11-expressing and INK128-treated HCCLM3 xenograft tumors were significantly smaller and less than the vector control, and were further reduced when SLFN11 expression was combined with INK128 treatment (**Figure 6B-C**). The increase in the size and weight in shSLFN11-expressing SMMC-7721 xenograft tumors (vs. control) was reduced by INK128 treatment (**Figure 6D-E**). Furthermore, the most significant reduction of size and weight of the SMMC-7721 xenograft tumors (relatively high SLFN11 expression) was observed with INK128 treatment alone (**Figure 6D-E**). Tumors with high SLFN11 expression (SLFN11-expressing HCCLM3) or with INK128 treatment had markedly suppressed levels of Ki-67, phospho-S6, and phospho-eIF4E (**Figure 6F**). The increased in Ki-67, phospho-S6, and phospho- eIF4E was attenuated in the SMMC-7721-shSLFN11 xenografts treated with INK128 (**Figure 6G**). Overall, the highest reduction of those markers was observed for the combination of SLFN11 expression and INK128 in HCCLM3 (low SLF11) xenograft tumors or INK128 alone in SMMC-7721 (high SLFN11) xenograft tumors (**Figure 6F-G**). In addition, overexpression of SLFN11 significantly reduced the lung metastatic nodules (**Figure 7A**). Likewise, INK128 could successfully attenuated the degree of lung metastasis induced by SLFN11 knockdown (**Figure 7B**). Consistent with the above observations, the most dramatic inhibition in lung metastasis was observed for SLFN11 expression and INK128 in combination for HCCLM3 and INK128 alone in SMMC-7721. Together, these results suggested that SLFN11 inhibits HCC tumorigenesis and metastasis by suppressing the mTOR signaling pathway and that SLFN11 may enhance HCC cell sensitivity to INK128.

## Discussion

Accumulating evidence indicates that the abnormal expression of oncogenes or tumor suppressors plays a vital role in the tumorigenesis and metastasis of HCC 27-29. As previously reported, HIV-1, a retrovirus, is inhibited by SLFN11 10. Considering that HCC in most patients in China and Asia is derived from infection of HBV, which is also a retrovirus, we sought to investigate the functions and pathways of SLFN11 in HCC. We found that SLFN11 expression was significantly downregulated in the tumor tissues of HCC compared with that expression in adjacent non-tumor tissues. Our results also demonstrated that SLFN11 expression was lower in HCC cell lines and provided a powerful capacity for predicting OS and RFS in HCC patients. Furthermore, *in vitro* and *in vivo* experiments showed that SLFN11 likely functions as a tumor suppressor in HCC progression and metastasis by targeting RPS4X via the mTOR signaling pathway. Therefore, our findings suggested that SLFN11 may act as a critical prognostic indicator to identify HCC patients with shorter OS and higher recurrence rates, and may provide new insight into novel therapies to improve the prognosis of HCC patients.

Previously, SLFN11 was reported to be a nuclear protein in NCI-H23 (Lung adenocarcinoma), DU-145 (Prostate cancer), and K562 (Chronic myelogenous leukemia) cell lines 11, 14, 30. However, in our study, we found that SLFN11 was prevalently stained in cytoplasmic of HCC tissues and cells. It was reported that v-slfn (a protein encoded by camelpox virus gene), which is homologous to mouse SLFNs, mainly located in cytoplasm, without obvious expression in nuclei or other organelles 31. In addition, SLFN5, a human protein of SLFN family, was reported to be a nuclear protein in melanoma 32. While in HCC tissues of Peng's study, SLFN5 was also mainly detected in cytoplasmic 33. Because the majority of our patients (about 85%) were infected with HBV and 2% were infected with HCV, we speculated that SLFN11 might have additional functions in cytoplasmic in HCC cells under specific condition such as under viral infection.

Studies on SLFN11 have so far primarily focused on its importance in drug sensitivity whereas the functions and pathways of SLFN11 itself in tumorigenesis and metastasis are less clear. Based on bioinformatics analyses associated with the Cancer Cell Line Encyclopedia (CCLE) and the National Cancer Institute Antitumor Cell Line Panel (NCI-60), SLFN11 appears to be a key factor in response to several anticancer drugs, including DNA synthesis inhibitors (gemcitabine, cytarabine), alkylating agents (chlorambucil, melphalan, cisplatin, carboplatin, carmustine), topoisomerase I inhibitors (topotecan, irinotecan), and topoisomerase II inhibitors (mitoxantrone, etoposide, daunorubicin, doxorubicin) 11, 26, 34. In Ewing sarcoma, the E26 transformation-specific (ETS) transcription factor (EWS-FLI1) is reported to drive SLFN11 to initiate a drug response 35. In addition, the downregulated RNA and protein expression of SLFN11 is associated with the hypermethylation of the *SLFN11* CpG promoter island 30. A recent study suggested that EZH2 facilitates chemoresistance by epigenetically suppressing SLFN11 12. However, we could not successfully restore the reduced SLFN11 expression in HCC cell lines by using 5-aza-2-deoxycytidine (data not shown). SLFN11 has also been shown to block replication independently of ATR (ataxia telangiectasia and Rad3-related protein) and bind resected DNA ends through replication protein A1 (RPA1) to enhance the drug sensitivity of cancer cells 14, 36, 37. We also showed INK128 treatment in mice harboring xenograft tumors with high SLFN11 expression led to a greater reduction in tumor size and metastasis compared with those with low SLFN11 expression. These data are consistent with the observations that SLFN11 enhances sensitivity to anticancer drugs. We hypothesized that INK128 will cause a negative feedback on SLFN11, and then the effect of INK128 will be diminished. However, the detailed mechanism about the synergistic effects between SLFN11 and INK128 is still unclear, which needs to be further investigated in the future.

Considering the significant role of the mTOR signaling pathway in many types of cancer 38-40, mTOR inhibitors may be effective in HCC patients with low SLFN11 expression. At present, many rapamycin analogs, such as temsirolimus and everolimus, have been approved by the Food and Drug Administration 41. However, rapamycin analogs have demonstrated limited efficacy for cancer treatment in the clinic, and this has been attributed to the partial inhibition of the 4E-BP1/eIF4E axis 42, 43. As previous studies reported, it was necessary for us to inhibit mTORC1 effectors (RPS6 and eIF4E) completely in hepatocarcinogenesis 44. Hsieh et al. reported that INK128 completely restored phosphorylation of the 4E-BP1/eIF4E and p70S6K/RPS6 axes in a prostate cancer preclinical study 45, which means that INK128 may have effects in HCC. Currently, INK128 is undergoing an international phase 1/2 clinical trial to determine its efficacy in patients with advanced or metastatic HCC (NCT02575339, ClinicalTrials.gov). Our findings demonstrated that INK128 successfully suppresses the mTOR signaling pathway and attenuates tumor growth and the degree of lung metastasis induced by low SLFN11 expression.

## Conclusions

In summary, we showed that SLFN11 was downregulated in HCC and acted as an independent prognostic factor for HCC patients. Functional assays demonstrated the significance of SLFN11 in inhibiting HCC tumorigenesis and metastasis by targeting RPS4X via the mTOR signaling pathway. Notably, INK128 may effectively suppress tumor progression and metastasis of HCC in patients with low SLFN11 expression (**Figure 7C**). These data suggested that SLFN11 may be a promising biomarker for HCC patients after hepatectomy and may offer a novel therapeutic strategy for HCC in the future. These encouraging preclinical results warrant validation in future clinical trials for HCC.

## Abbreviations

HCC: hepatocellular carcinoma
SLFN11: Schlafen family member 11
RPS4X: ribosomal protein S4 X-linked
HIV-1: human immunodeficiency virus 1
HBV: hepatitis B virus
DDAs: DNA-damaging agents
TMA: tissue microarray
shRNA: small hairpin RNA
OS: overall survival
RFS: recurrence- free survival
qRT-PCR: quantitative reverse transcription-polymerase chain reaction
BrdU: 5- bromo-2'-deoxyuridine
IHC: immunohistochemical
CCK-8: Cell Counting Kit-8
ROC: receiver operating characteristic
AFP: alpha-fetoprotein
BCLC: Barcelona Clinic Liver Cancer
TNM: tumor-nodes- metastasis
IP: immunoprecipitation
LC-MS/MS: liquid chromatography coupled with tandem mass spectrometry
OE: overexpressing
KD: knockdown
HR: hazard ratio
CI: confidence interval
EIF2: eukaryotic initiation factor 2
eIF4: eukaryotic initiation factor 4
eIF4E: eukaryotic initiation factor 4E
S6K: ribosomal protein S6 kinase
RPS6: ribosomal protein S6
p-: phosphorylated
Bax: BCL2-associated X protein
Bak: BCL2 Antagonist/Killer 1
Bcl-2: BCL2 Apoptosis Regulator
