# Supplementary material for: SLFN11 inhibits hepatocellular carcinoma tumorigenesis and metastasis by targeting RPS4X via mTOR pathway
Source: Theranostics. 2020 Mar 25;10(10):4627–43. doi: 10.7150/thno.42869 (PMC7150495; doi:10.7150/thno.42869)
Supplement: Supplementary file 1 — Supplementary figures and tables. [file thnov10p4627s1.pdf]

Supplementary Material

SLFN11 inhibits hepatocellular carcinoma tumorigenesis and metastasis by targeting RPS4X via mTOR pathway

Chenhao Zhou[#], Chunxiao Liu[#], Wenjie Liu[#], Wanyong Chen, Yirui Yin, Chia-Wei Li, Jennifer L. Hsu, Jialei Sun, Qiang Zhou, Hui Li, Bo Hu, Peiyao Fu, Manar Atyah, Qianni Ma, Yang Xu, Qiongzhu Dong[*], Mien-Chie Hung[*], and Ning Ren[*]

* Correspondence to: ren.ning@zs-hospital.sh.cn;

mhung@cmu.edu.tw;

qzhdong@fudan.edu.cn

[#] The authors contributed equally to this work.

Supplementary Material includes:

Supplementary Methods

Supplementary Figure S1 to Figure S6

Supplementary Table S1 to Table S5

Supplementary Methods

Cell transfection

The lentiviral-based small hairpin RNA (shRNA) targeting SLFN11 or RPS4X and SLFN11 overexpression lentiviruses were constructed by Hanyin Biotechnology Co., Ltd., Shanghai, China. They also provided the control lentivirus with shRNA (Control) and plasmid (Vector). HCC cells were cultured in six-well plates overnight and infected with the corresponding lentiviruses in the presence of polybrene (8 μg/ml). After the cells were infected for 24 h, the original medium was replaced by fresh 10% FBS DMEM supplemented with puromycin (3 μg/ml) for excluding noninfected HCC cells. The following experiments were performed by using the successfully selected HCC cell lines. The target sequences of sh1-SLFN11 were 5'-CAGTCTTTGAGAGAGCTTATT-3', sh2-SLFN11 was 5'-GCTCAGAATTTCCGTACTGAA-3', and shRPS4X was 5'- TGACAAGACGGGAGAGAAT-3'.

RNA extraction and quantitative reverse transcription-polymerase chain reaction (qRT-PCR)

TRIzol reagent (Invitrogen, USA) was used to extract total RNA of frozen tissues of patients and cells enrolled in our study. Then a PrimeScript RT reagent kit (Takara, Japan) was used for the complementary DNA synthesis reactions. According to the manufacturer's protocol, SYBR® Premix ExTaq™ (Takara, Japan) was used to perform the qRT-PCR in an ABI Prism 7500 Sequence Detection System (Applied Biosystems, Foster City, CA, USA). The primers designed in our study were as follows: SLFN11, forward: 5'-CCTGGTTGTGGAACCATCTT-3', and reverse: 5'-CTCTCCTTCTCTTGGTCTCTCT-3'; GAPDH, forward: 5'-CTGGGCTACACTGAGCACC-3', and reverse: 5'-AAGTGGTCGTTGAGGGCAATG-3'; RPS4X, forward: 5'-AGATTTGCATGCAGCGGTTC-3', and reverse: 5'-

GGCCTCCTCAGGTGTAATACG-3'. The results were normalized to GAPDH for measuring the relative mRNA expression. Triplicate experiments were performed in each sample.

Western blot analysis

Total proteins of frozen tissues and cells were extracted by using RIPA buffer (150 mM NaCl, 50 mM Tris [pH7.5], 0.1% sodium dodecyl sulfate (SDS), 1% Triton X-100, 0.5% sodium deoxycholate) supplemented with 1% protease inhibitor cocktail and phosphatase inhibitor cocktail (Bimake, Houston, TX, USA). For drug treatment assays, we treated cells with INK128 (200 nM; SelleckChem, Shanghai, China) for 48 h. Then we extracted the proteins from the cells. The proteins were then quantified by the Pierce™ BCA Protein Assay Kit (Pierce Biotechnology, Rockford, IL, USA). Total protein (20 μg) was heated for 15 min at 100 °C, then separated by SDS-polyacrylamide gel electrophoresis (SDS-PAGE) and transferred to polyvinylidene difluoride (PVDF) membranes (Millipore, Billerica, MA, USA). 5% nonfat dry milk was used to block the membranes for 1 hour at room temperature; then the corresponding primary antibodies were incubated on the membranes at 4 °C overnight. After the membranes were washed three times by Tris-buffered saline-Tween (TBST) solution, the horseradish peroxidase (HRP)-conjugated secondary antibody (Jackson ImmunoResearch, West Grove, PA, USA) with 1:5000 dilutions were incubated on the membranes at room temperature for 1 h. An enhanced chemiluminescence (ECL) assay (Pierce Biotechnology) was used for bands visualization, and the bands were then exposed to ImageQuant™ LAS 4000 (GE Healthcare, Pittsburgh, PA, USA).

Mouse xenograft study

All male BALB/c nude mice (4–6 weeks of age) were purchased from the Shanghai SLAC

Laboratory Animal Co., Ltd., and raised in a standard pathogen-free (SPF) environment in the experimental animal center of Zhongshan Hospital, Fudan University. Our project was conducted under guidelines approved by the Institutional Animal Care and Ethics Committee of Zhongshan Hospital, Fudan University. For the subcutaneous mouse models, 5×10^6 HCC cells (HCCLM3-Vector, HCCLM3-SLFN11, SMMC-7721-Control, SMMC-7721-shSLFN11) that had been suspended in 100 μl of PBS solution were subcutaneously injected into the right flanks of the mice (6/group). The mice were monitored every day, and the subcutaneous tumors were measured by a vernier caliper twice a week. Six weeks later, all mice were euthanized. Tumor volume was calculated as follows: Tumor volume (mm^3) = (Length × Width^2)/2. For the orthotopic mouse models, tumors from subcutaneous HCC cells (HCCLM3-Vector, HCCLM3-SLFN11, SMMC-7721-Control, SMMC-7721-shSLFN11) were cut into 1 mm^3 cubes and transplanted to the liver parenchyma of BALB/c nude mice that had been anesthetized with use of isoflurane. After the cells had been implanted for 1 week, the mice were randomly divided into 8 groups (HCCLM3-Vector, HCCLM3-Vector + INK128, HCCLM3-SLFN11, HCCLM3-SLFN11+ INK128, SMMC-7721-Control, SMMC-7721-Control + INK128, SMMC-7721-shSLFN11, SMMC-7721-shSLFN11+ INK128). For drug administration, INK128 was dissolved in 30% PEG400, 0.5% Tween80, and 5% propylene glycol. INK128 was administrated by oral gavage at 1 mg/kg/day. For the HCCLM3-Vector and SMMC-7721-Control group, the mice were treated via oral gavage with vehicle daily. At six weeks after transplantation, all mice were euthanized. The subcutaneous tumors or the orthotopic tumors in livers were excised, photographed, measured, and weighed. The xenograft tumors were fixed in 4% paraformaldehyde and embedded in paraffin for further IHC staining. In addition, we harvested the lung tissues of all orthotopic mouse models to evaluate the incidence of lung metastasis. They were also fixed in 4% paraformaldehyde and embedded in

paraffin. Then the embedded blocks received consecutive resection for further hematoxylin and eosin (HE) staining. Lung metastases were evaluated by two experienced pathologists.

Supplementary Figures and Figure Legends

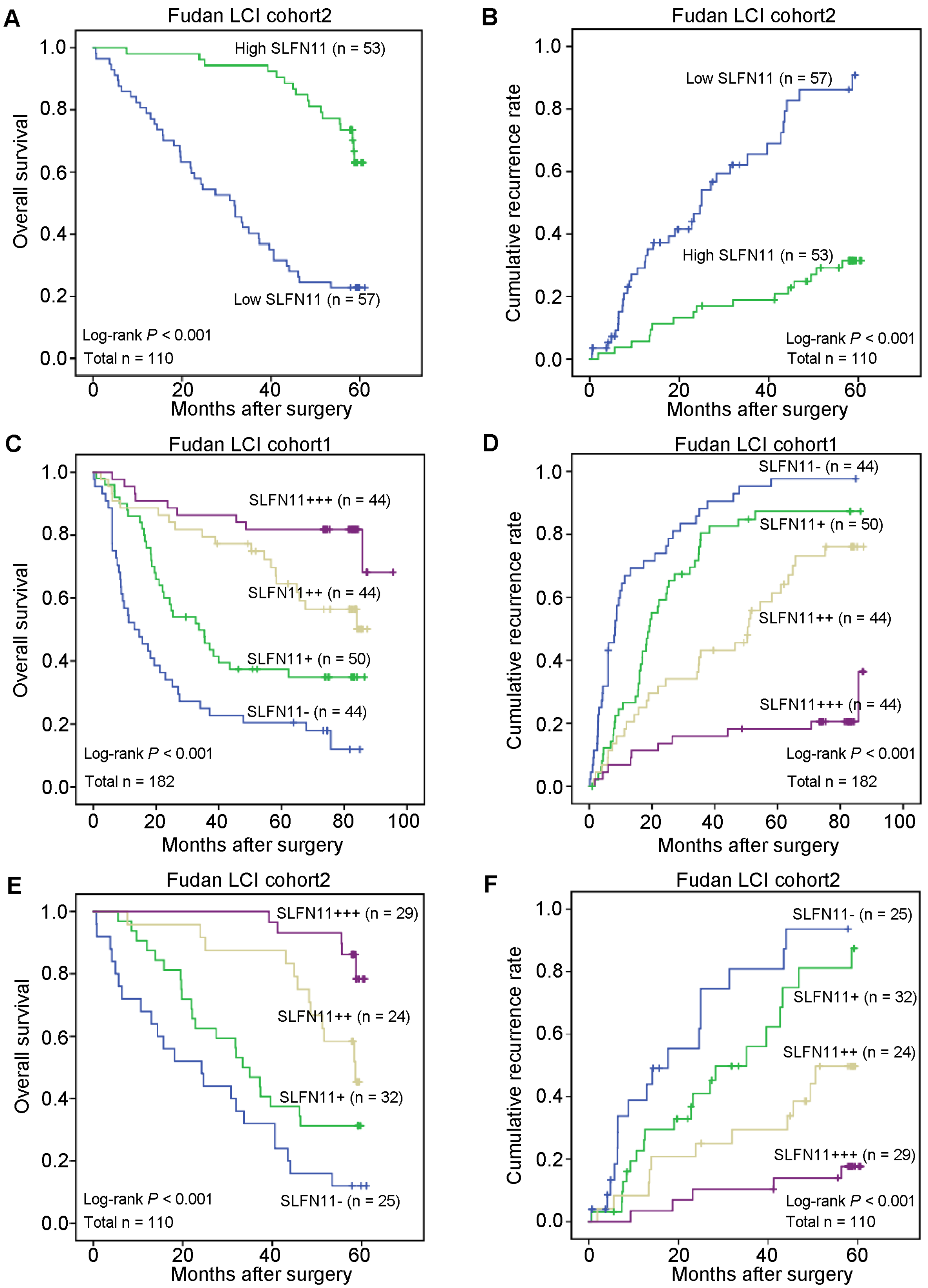

Figure S1. (**A)** Kaplan-Meier curves for overall survival based on SLFN11 expression in Fudan LCI cohort 2. (**B**) Kaplan-Meier curves for recurrence-free survival based on SLFN11 expression in the Fudan LCI cohort 2. (**C, D**) OS and recurrence probability of patients in Fudan LCI cohort 1 based on the status of SLFN11: negative (–), weak (+), moderate (++), and strong (+++). (**E, F**) OS and recurrence probability of patients in Fudan LCI cohort 2 based on the status of SLFN11: negative (–), weak (+), moderate (++), and strong (+++).

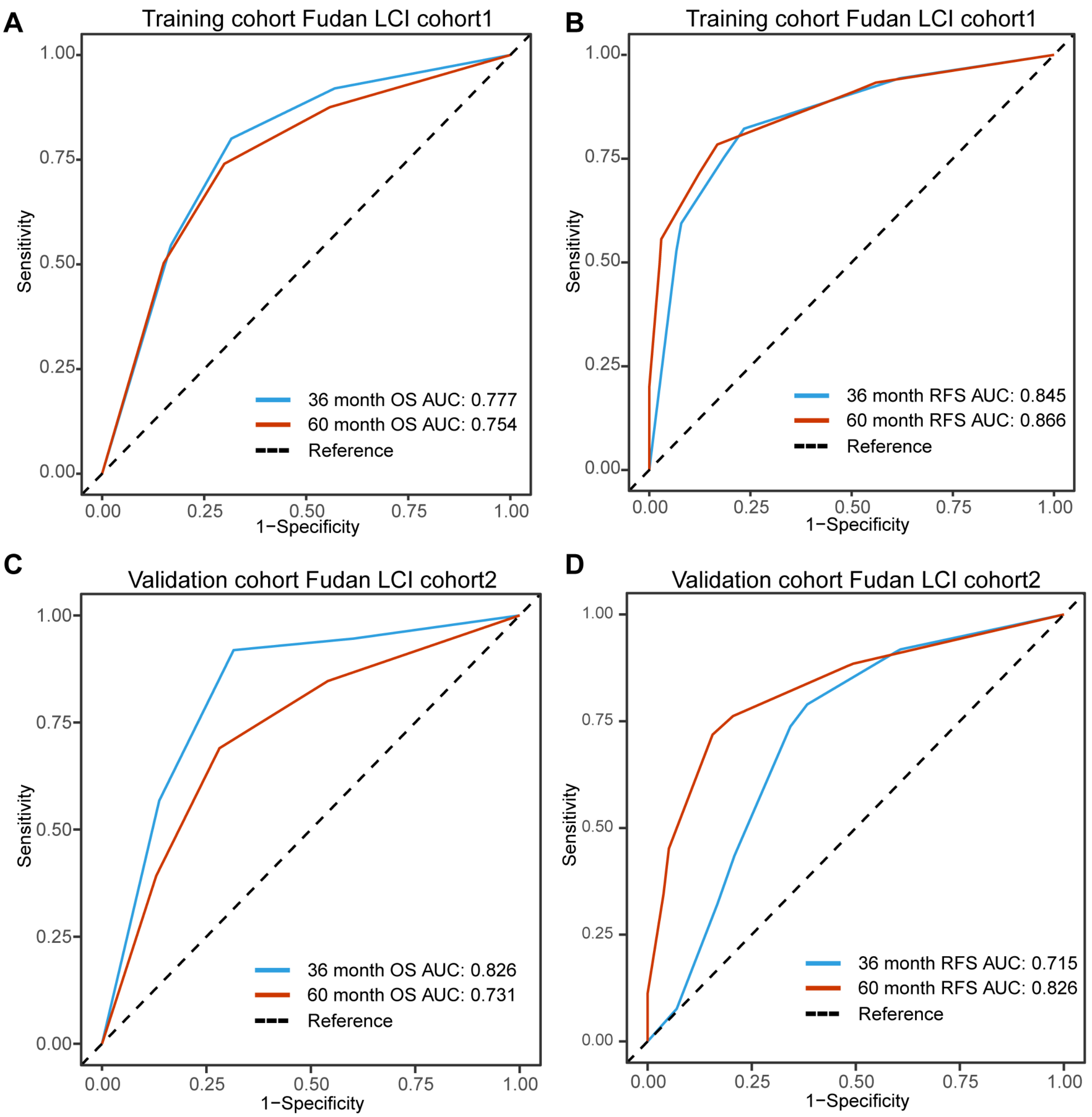

Figure S2. Time-dependent receiver operating characteristic (ROC) analyses for the OS and RFS prediction were performed based on the multivariable models containing SLFN11 to signify the accuracy of SLFN11 expression as an adjunct for biomarker analysis. (**A**) OS prediction in training cohort, (**B**) RFS prediction in training cohort, (**C**) OS prediction in validation cohort, (**D**) RFS prediction in validation cohort.

Figure S3

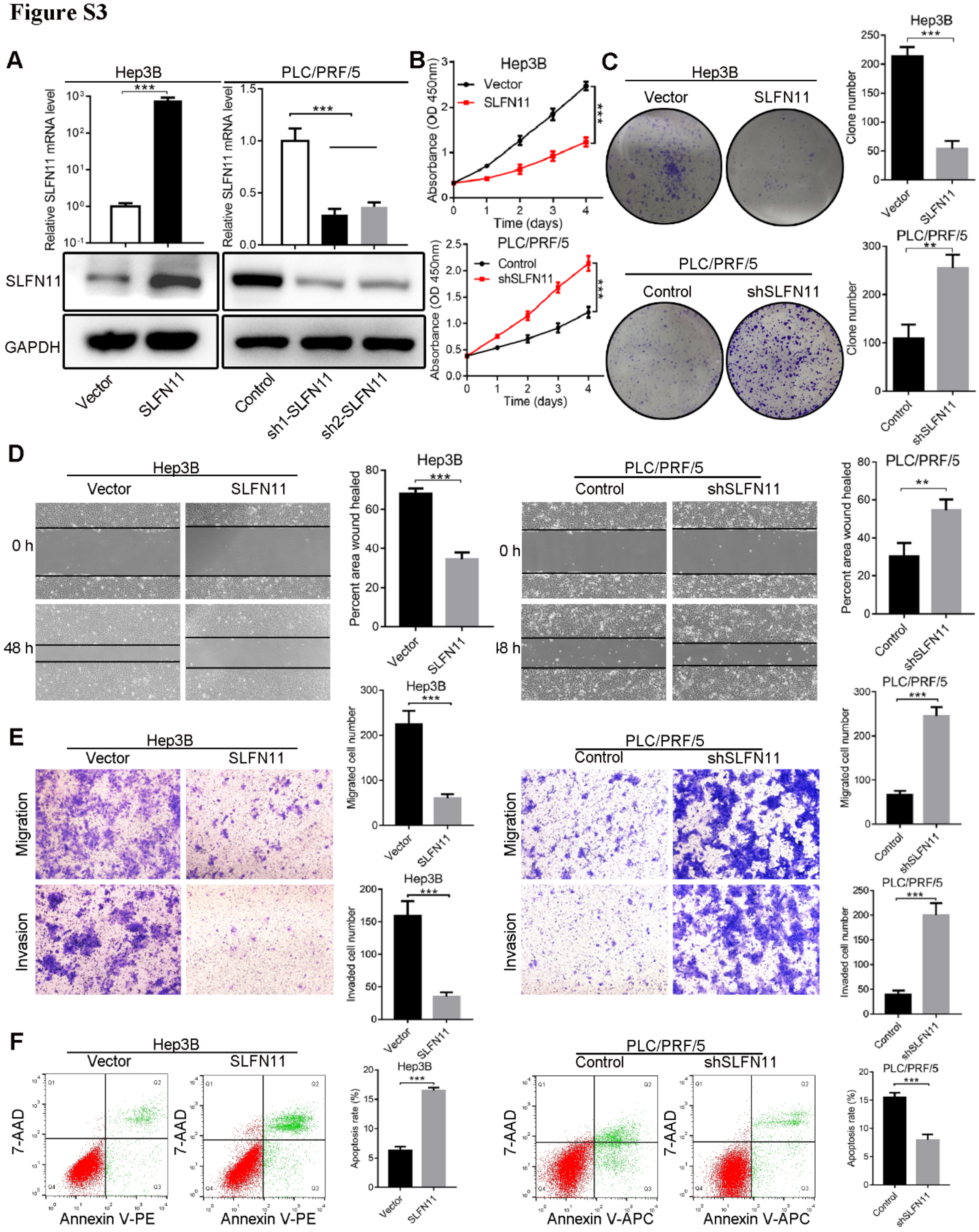

Figure S3. SLFN11 inhibits cell proliferation, migration, and invasion and facilitates apoptosis *in vitro*. (A) Overexpression and knockdown efficiency of SLFN11 was verified by

qRT-PCR and Western blot assays in Hep3B and PLC/PRF/5 cells. **(B)** Effects of SLFN11 overexpression and knockdown on cell proliferation by CCK-8 in Hep3B and PLC/PRF/5 cells. **(C)** Effects of SLFN11 overexpression and knockdown on cell proliferation by colony formation assays in Hep3B and PLC/PRF/5 cells. **(D)** Effects of SLFN11 overexpression and knockdown on cell migratory abilities by wound healing assays in Hep3B and PLC/PRF/5 cells. **(E)** Effects of SLFN11 overexpression and knockdown on cell migratory and invasive capacities by transwell assays in Hep3B and PLC/PRF/5 cells. **(F)** Effects of SLFN11 overexpression and knockdown on cell apoptosis by flow cytometry in Hep3B and PLC/PRF/5 cells. * $P < 0.05$, ** $P < 0.01$, *** $P < 0.001$.

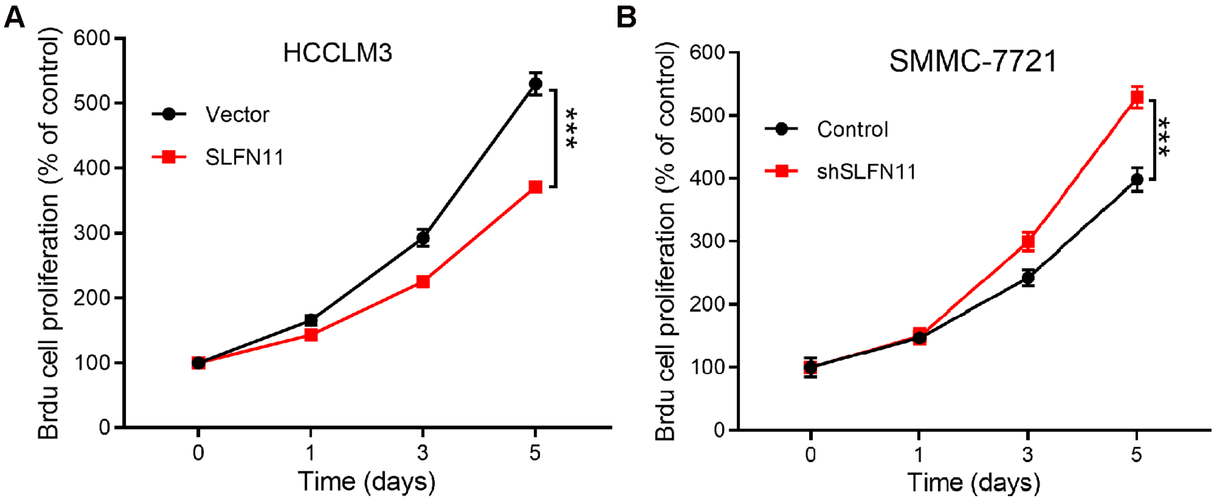

Figure S4 The incorporated Brdu of HCCLM3 (A) and SMMC-7721 (B) cells were detected by Brdu cell proliferation assay kit for 5 days. *** $P < 0.001$.

Figure S5

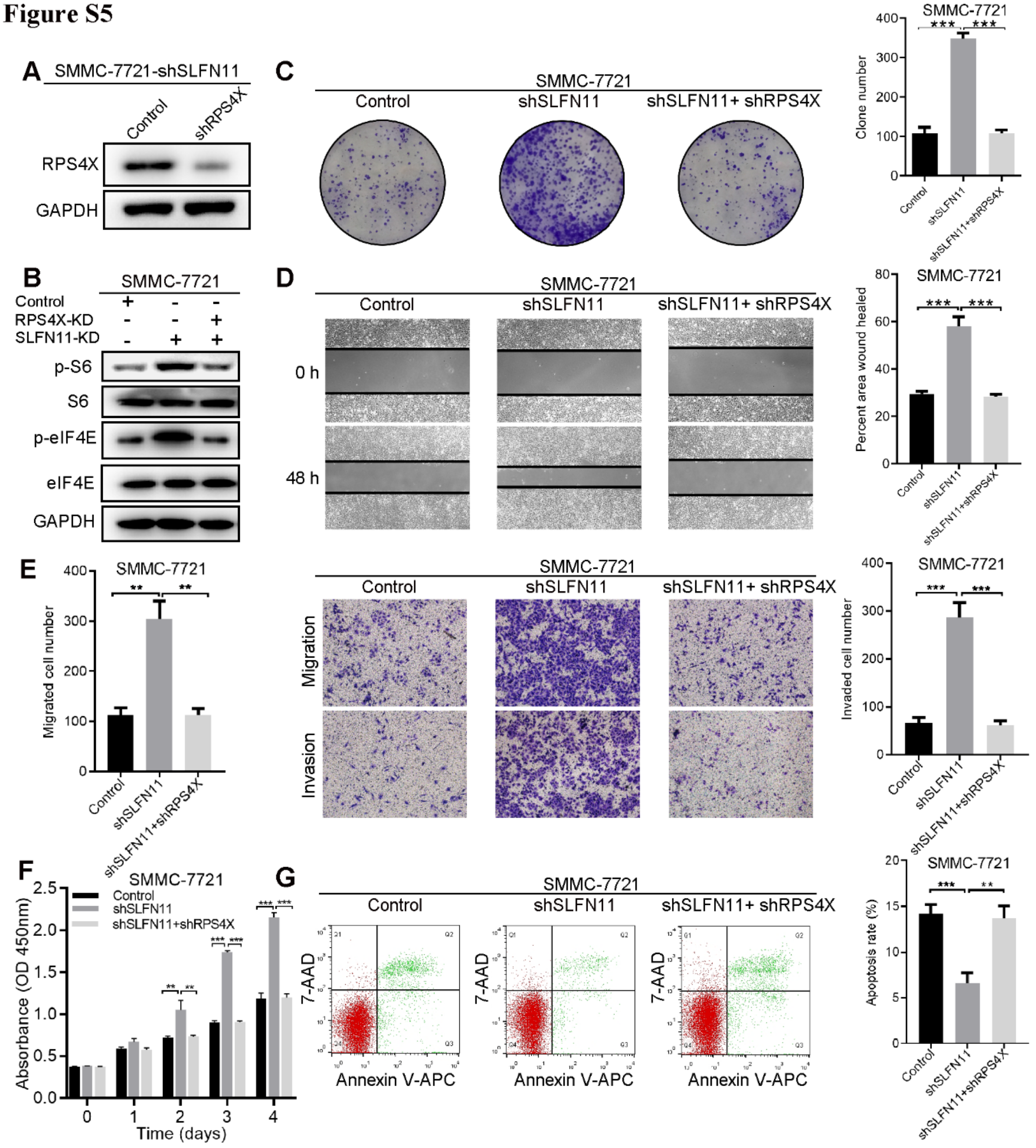

Figure S5. SLFN11 knockdown promotes mTOR signaling pathway and HCC progression via RPS4X. (A) Western blot analysis of the knockdown efficiency of RPS4X in SMMC-7721-shSLFN11 cells. **(B)** Western blot shows that RPS4X knockdown restored increased phosphorylation of S6 and eIF4E in SMMC-7721-shSLFN11 cells. **(C)** Colony formation assays were conducted to study the cell proliferation of SMMC-7721-Control cells and SMMC-7721-

shSLFN11 cells with or without RPS4X knockdown. **(D)** Wound healing assays were performed to detect the cell migratory abilities of SMMC-7721-Control cells and SMMC-7721-shSLFN11 cells with or without RPS4X knockdown. **(E)** Transwell assays were used to investigate the cell migratory and invasive capacities of SMMC-7721-Control cells and SMMC-7721-shSLFN11 cells with or without RPS4X knockdown. **(F)** CCK-8 assays were conducted to determine the cell proliferation of SMMC-7721-Control cells and SMMC-7721-shSLFN11 cells with or without RPS4X knockdown. **(G)** Cell apoptosis was assessed by flow cytometry in SMMC-7721-Control cells and SMMC-7721-shSLFN11 cells with or without RPS4X knockdown. ** $P < 0.01$, *** $P < 0.001$.

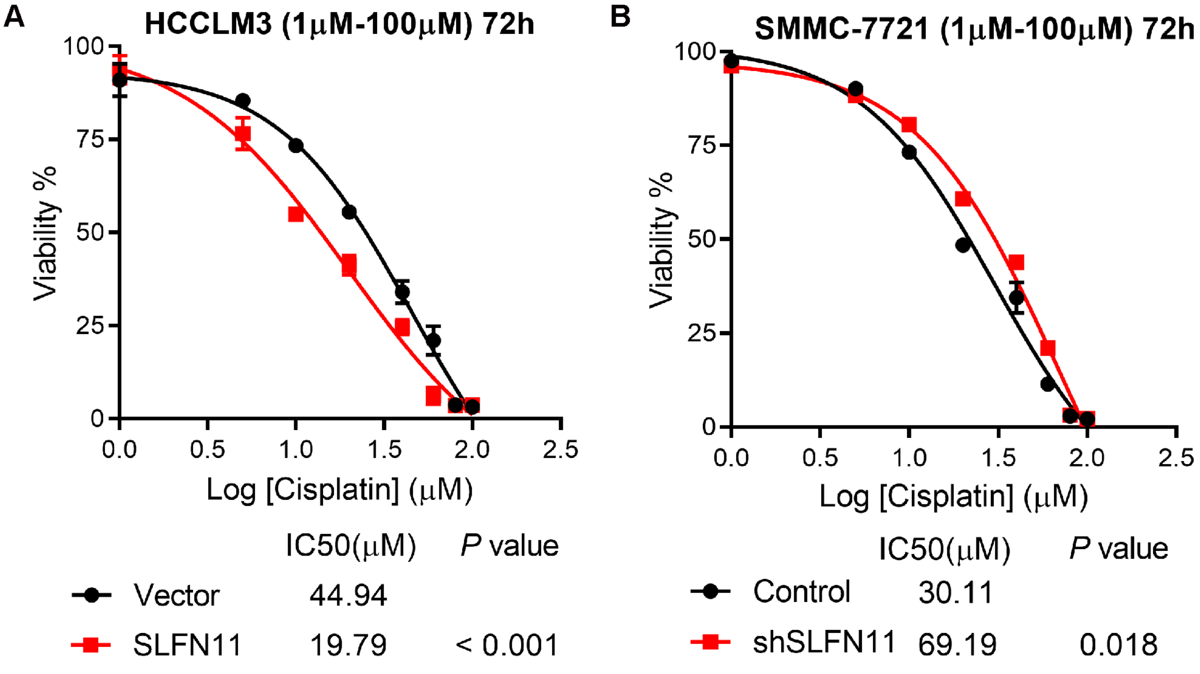

Figure S6. Impact of SLFN11 overexpression (A) or knockdown (B) in cisplatin chemo-resistance of HCC. Cell viability was determined by CCK-8 assay following exposure to cisplatin. The corresponding half-maximal inhibitory concentration (IC50) values and *P* values were also shown.

Table S1. Correlation between tumor SLFN11 expression and clinicopathologic characteristics in cohort 1

**Characteristics**		**Patients**	**Tumor SLFN11 expression**		***P* value**
		**Number (%)**	**Low (n = 94)**	**High (n = 88)**	
Age, years	≤ 50	79 (43.4)	44	35	0.371
	> 50	103 (56.6)	50	53	
Gender	Female	22 (12.1)	11	11	1.000
	Male	160 (87.9)	83	77	
HBsAg	Negative	29 (15.9)	14	15	0.840
	Positive	153 (84.1)	80	73	
HCV	Negative	178 (97.8)	93	85	0.355
	Positive	4 (2.2)	1	3	
AFP, ng/ml	≤ 20	67 (36.8)	23	44	**< 0.001**
	> 20	115 (63.2)	71	44	
CEA, ng/ml	≤ 5	167 (91.8)	88	79	0.423
	> 5	15 (8.2)	6	9	
CA19-9, U/ml	≤ 36	139 (76.4)	68	71	0.223
	> 36	43 (23.6)	26	17	
ALT, U/L	≤ 40	105 (57.7)	54	51	1.000
	> 40	77 (42.3)	40	37	
AST, U/L	≤ 37	128 (70.3)	63	65	0.334
	> 37	54 (29.7)	31	23	
γ-GT , U/L	≤ 54	74 (40.7)	33	41	0.132
	> 54	108 (59.3)	61	47	
Ascites	Absent	173 (95.1)	90	83	0.741
	Present	9 (4.9)	4	5	
Tumor number	Single	152 (83.5)	77	75	0.690
	Multiple	30 (16.5)	17	13	
Tumor size, cm	≤ 5	87 (47.8)	35	52	**0.005**
	> 5	95 (52.2)	59	36	
Tumor encapsulation	Complete	101 (55.5)	49	52	0.373
	None	81 (44.5)	45	36	
Tumor differentiation	I-II	114 (62.6)	54	60	0.168
	III-IV	68 (37.4)	40	28	
Microvascular invasion	Absent	104 (57.1)	46	58	**0.025**
	Present	78 (42.9)	48	30	
BCLC stage	0+A	90 (49.5)	39	51	**0.038**
	B+C	92 (50.5)	55	37	
TNM stage	I+II	133 (73.1)	62	71	**0.030**
	III+IV	49 (26.9)	32	17	

Abbreviations: HCC, hepatocellular carcinoma; HBsAg, hepatitis B surface antigen; HCV, hepatitis C virus; AFP, α-fetoprotein; CEA: carcinoembryonic antigen; CA19-9: carbohydrate antigen 19-9; ALT: alanine aminotransferase; AST: aspartate aminotransferase; γ-GT, γ-glutamyl transferase; BCLC, Barcelona Clinic Liver Cancer; TNM, tumor-nodes-metastasis. * *P* value < 0.05 was considered statistically significant. *P* values were calculated using the Pearson chi-square test.

Table S2. Correlation between tumor SLFN11 expression and clinicopathologic characteristics in cohort 2

**Characteristics**		**Patients**	**Tumor SLFN11 expression**		***P* value**
		**Number (%)**	**Low (n = 57)**	**High (n = 53)**	
Age, years	≤ 50	34 (30.9)	18	16	1.000
	> 50	76 (69.1)	39	37	
Gender	Female	12 (10.9)	6	6	1.000
	Male	98 (89.1)	51	47	
HBsAg	Negative	17 (15.5)	8	9	0.793
	Positive	93 (84.5)	49	44	
HCV	Negative	108 (98.2)	56	52	1.000
	Positive	2 (1.8)	1	1	
AFP, ng/ml	≤ 20	55 (50.0)	22	33	**0.022**
	> 20	55 (50.0)	35	20	
CEA, ng/ml	≤ 5	98 (89.1)	48	50	0.127
	> 5	12 (10.9)	9	3	
CA19-9, U/ml	≤ 36	91 (82.7)	41	50	**0.002**
	> 36	19 (17.3)	16	3	
ALT, U/L	≤ 40	72 (65.5)	39	33	0.550
	> 40	38 (34.5)	18	20	
AST, U/L	≤ 37	66 (60.0)	34	32	1.000
	> 37	44 (40.0)	23	21	
γ-GT , U/L	≤ 54	60 (54.5)	31	29	1.000
	> 54	50 (45.5)	26	24	
Ascites	Absent	102 (92.7)	52	50	0.718
	Present	8 (7.3)	5	3	
Tumor number	Single	89 (80.9)	43	46	0.151
	Multiple	21 (19.1)	14	7	
Tumor size, cm	≤ 5	57 (51.8)	26	31	0.188
	> 5	53 (48.2)	31	22	
Tumor encapsulation	Complete	74 (67.3)	38	36	1.000
	None	36 (32.7)	19	17	
Tumor differentiation	I-II	66 (60.0)	31	35	0.246
	III-IV	44 (40.0)	26	18	
Microvascular invasion	Absent	72 (65.5)	32	40	**0.045**
	Present	38 (34.5)	25	13	
BCLC stage	0+A	59 (53.6)	24	35	**0.014**
	B+C	51 (46.4)	33	18	
TNM stage	I+II	88 (80.0)	41	47	**0.033**
	III+IV	22 (20.0)	16	6	

Abbreviations: HCC, hepatocellular carcinoma; HBsAg, hepatitis B surface antigen; HCV, hepatitis C virus; AFP, α-fetoprotein; CEA: carcinoembryonic antigen; CA19-9: carbohydrate antigen 19-9; ALT: alanine aminotransferase; AST: aspartate aminotransferase; γ-GT, γ-glutamyl transferase; BCLC, Barcelona Clinic Liver Cancer; TNM, tumor-nodes-metastasis. * *P* value < 0.05 was considered statistically significant. *P* values were calculated using the Pearson chi-square test.

Table S3. Univariate and multivariate analysis of factors associated with survival and recurrence in cohort 1

Variables	OS			RFS		
	Univariate	Multivariate		Univariate	Multivariate	
	P	HR (95% CI)	*P*	*P*	HR (95% CI)	*P*
Age, years (> 50 vs. ≤ 50)	0.545		NA	0.982		NA
Gender (male vs. female)	0.335		NA	0.089		NA
HBsAg (positive vs.negative)	0.266		NA	0.479		NA
AFP, ng/ml (> 20 vs. ≤ 20)	**0.003**		NS	**0.002**		NS
CEA, ng/ml (> 5 vs. ≤ 5)	0.388		NA	0.193		NA
CA19-9, U/ml (> 36 vs. ≤ 36)	**0.039**		NS	**0.002**		NS
ALT, U/L (> 40 vs. ≤ 40)	0.369		NA	0.052		NA
AST, U/L (> 37 vs. ≤ 37)	**0.006**		NS	**0.015**		NS
γ-GT , U/L (> 54 vs. ≤ 54)	**0.001**		NS	**< 0.001**	**1.712 (1.132-2.590)**	**0.011**
Ascites (present vs. absent)	0.118		NA	**0.043**		NS
Tumor number (multiple vs. single)	0.728		NA	0.251		NA
Tumor size, cm (> 5 vs. ≤ 5)	**< 0.001**	**1.682 (1.072-2.638)**	**0.024**	**< 0.001**		NS
Tumor encapsulation (complete vs. none)	0.070		NA	0.076		NA
Tumor differentiation (III–IV vs. I-II)	**0.023**		NS	0.062		NA
Microvascular invasion (present vs. absent)	**0.019**		NS	0.080		NA
BCLC stage (B+C vs. 0+A)	0.171		NA	0.237		NA
TNM stage (III+IV vs. I+II)	**< 0.001**		NS	**< 0.001**	**1.552 (1.037-2.324)**	**0.033**
SLFN11 (Low vs. High)	**< 0.001**	**3.142 (1.957-5.045)**	**< 0.001**	**< 0.001**	**3.659 (2.414-5.547)**	**< 0.001**
Subgroup analyses (SLFN11+++ as referent)						

SLFN11 - vs. SLFN11+++	**< 0.001**	**8.218 (3.892-17.354)**	**< 0.001**	**< 0.001**	**16.305 (7.982-33.305)**	**< 0.001**
SLFN11 + vs. SLFN11+++	**< 0.001**	**4.294 (2.012-9.162)**	**< 0.001**	**< 0.001**	**8.475 (4.189-17.146)**	**< 0.001**
SLFN11 ++ vs. SLFN11+++	**0.030**	**2.279 (1.021-5.090)**	**0.044**	**< 0.001**	**4.516 (2.203-9.259)**	**< 0.001**

Abbreviations: OS, overall survival; RFS recurrence-free survival; HBsAg, hepatitis B surface antigen; AFP: α-fetoprotein; CEA: carcinoembryonic antigen; CA19-9: carbohydrate antigen 19-9; ALT: alanine aminotransferase; AST: aspartate aminotransferase; γ-GT, γ-glutamyl transferase; HR, hazard ratio; CI, confidential interval; NA, not adopted; NS, not significant. Data obtained from the Cox proportional hazards model; *P* value < 0.05 was regarded as statistically significant.

Table S4. Univariate and multivariate analysis of factors associated with survival and recurrence in cohort 2.

Variables	OS			RFS		
	Univariate	Multivariate		Univariate	Multivariate	
	P	HR (95% CI)	*P*	*P*	HR (95% CI)	*P*
Age, years (> 50 vs. ≤ 50)	0.851		NA	0.412		NA
Gender (male vs. female)	0.353		NA	0.349		NA
HBsAg (positive vs.negative)	0.809		NA	0.673		NA
AFP, ng/ml (> 20 vs. ≤ 20)	**0.011**		NS	**0.031**		NS
CEA, ng/ml (> 5 vs. ≤ 5)	0.825		NA	0.394		NA
CA19-9, U/ml (> 36 vs. ≤ 36)	**0.002**	**2.856 (1.364-5.980)**	**0.005**	0.109		NA
ALT, U/L (> 40 vs. ≤ 40)	**0.019**	**2.864 (1.460-5.618)**	**0.002**	0.530		NA
AST, U/L (> 37 vs. ≤ 37)	0.499		NA	0.333		NA
γ-GT , U/L (> 54 vs. ≤ 54)	0.713		NA	0.786		NA
Ascites (present vs. absent)	0.353		NA	0.965		NA
Tumor number (multiple vs. single)	0.057		NA	0.299		NA
Tumor size, cm (> 5 vs. ≤ 5)	0.114		NA	0.395		NA
Tumor encapsulation (complete vs. none)	0.125		NA	0.665		NA
Tumor differentiation (III–IV vs. I-II)	0.976		NA	0.222		NA
Microvascular invasion (present vs. absent)	0.134		NA	**0.024**		NS
BCLC stage (B+C vs. 0+A)	**0.037**		NS	0.106		NA
TNM stage (III+IV vs. I+II)	**0.003**		NS	0.853		NA
SLFN11 (Low vs. High)	**< 0.001**	**3.924 (2.169-7.099)**	**< 0.001**	**< 0.001**	**4.977 (2.639-9.388)**	**< 0.001**
Subgroup analyses (SLFN11+++ as referent)						

SLFN11 - vs. SLFN11+++	**< 0.001**	**14.384 (5.197-39.813)**	**< 0.001**	**< 0.001**	**14.545 (5.148-41.091)**	**< 0.001**
SLFN11 + vs. SLFN11+++	**< 0.001**	**5.753 (2.094-15.807)**	**0.001**	**< 0.001**	**7.685 (2.755-21.440)**	**< 0.001**
SLFN11 ++ vs. SLFN11+++	**0.023**	**4.156 (1.424-12.125)**	**0.009**	**0.022**	**3.287 (1.127-9.591)**	**0.029**

Abbreviations: OS, overall survival; RFS recurrence-free survival; HBsAg, hepatitis B surface antigen; AFP: α-fetoprotein; CEA: carcinoembryonic antigen; CA19-9: carbohydrate antigen 19-9; ALT: alanine aminotransferase; AST: aspartate aminotransferase; γ-GT, γ-glutamyl transferase; HR, hazard ratio; CI, confidential interval; NA, not adopted; NS, not significant. Data obtained from the Cox proportional hazards model; *P* value < 0.05 was regarded as statistically significant.

Table S5. The list of 84 unique proteins that interact with SLFN11 by LC-MS/MS analyses.

Entry name	Description	Protein score
IGKV2-30	Immunoglobulin kappa variable 2-30	835
SLFN11	Schlafen family member 11	304
KRT6B	Keratin, type II cytoskeletal 6B	211
HNRNPU	Heterogeneous nuclear ribonucleoprotein U	196
DHX9	ATP-dependent RNA helicase A	133
RPL13	60S ribosomal protein L13	122
RBM10	RNA-binding protein 10	73
PRPF31	U4/U6 small nuclear ribonucleoprotein Prp31	65
PGAM5	Serine/threonine-protein phosphatase PGAM5,mitochondrial	64
TXN	Thioredoxin	58
RPL15	60S ribosomal protein L15	56
USP15	Ubiquitin carboxyl-terminal hydrolase 15	54
SPTAN1	Spectrin alpha chain, non-erythrocytic 1	53
RPL14	60S ribosomal protein L14	49
KRT75	Keratin, type II cytoskeletal 75	49
MATR3	Matrin-3	48
RPL31	60S ribosomal protein L31	46
MRPL37	39S ribosomal protein L37, mitochondrial	46
STK38L	Serine/threonine-protein kinase 38-like	46
EPRS	Bifunctional glutamate/proline--tRNA ligase	44
EXOSC3	Exosome complex component RRP40	43
PEX6	Peroxisome assembly factor 2	43
RPS3A	40S ribosomal protein S3a	43
HNRNPA2B1	Heterogeneous nuclear ribonucleoproteins A2/B1	42
ALYREF	THO complex subunit 4	41
MRPL58	Peptidyl-tRNA hydrolase ICT1, mitochondrial	40
EFTUD2	116 kDa U5 small nuclear ribonucleoprotein component	39
MRPL2	39S ribosomal protein L2, mitochondrial	39
RPS11	40S ribosomal protein S11	38
MRPL45	39S ribosomal protein L45, mitochondrial	38
RPS4X	40S ribosomal protein S4, X isoform	37
SLC25A3	Phosphate carrier protein, mitochondrial	37
LCN1P1	Putative lipocalin 1-like protein 1	37
MRPL15	39S ribosomal protein L15, mitochondrial	37
RPL7A	60S ribosomal protein L7a	36
PPM1B	Protein phosphatase 1B	36
DHX30	Putative ATP-dependent RNA helicase DHX30	35
IGKV4-1	Immunoglobulin kappa variable 4-1	35
NONO	Non-POU domain-containing octamer-binding protein	34
FMR1	Synaptic functional regulator FMR1	33
MRPL19	39S ribosomal protein L19, mitochondrial	33
MRPL43	39S ribosomal protein L43, mitochondrial	33
SNRPD2	Small nuclear ribonucleoprotein Sm D2	32
SBNO2	Protein strawberry notch homolog 2	32
KCTD7	BTB/POZ domain-containing protein KCTD7	32
ELAVL1	ELAV-like protein 1	32
NAT10	RNA cytidine acetyltransferase	31
RPS25	40S ribosomal protein S25	31
TAF4	Transcription initiation factor TFIID subunit 4	30
DDX5	Probable ATP-dependent RNA helicase DDX5	30

STRN4	Striatin-4	30
SLC25A5	ADP/ATP translocase 2	30
ATP5A1	ATP synthase subunit alpha, mitochondrial	29
RPS19	40S ribosomal protein S19	29
NOP2	Probable 28S rRNA (cytosine(4447)-C(5))-methyltransferase	29
CLNS1A	Methylosome subunit pICln	28
FAM196A	Protein FAM196A	27
OTUD4	OTU domain-containing protein 4	27
RPL3	60S ribosomal protein L3	27
SRSF4	Serine/arginine-rich splicing factor 4	27
THEMIS	Protein THEMIS	27
CABP4	Calcium-binding protein 4	27
STK17A	Serine/threonine-protein kinase 17A	26
MRPL9	39S ribosomal protein L9, mitochondrial	26
TOP2A	DNA topoisomerase 2-alpha	26
RUVBL2	RuvB-like 2	26
URAD	Putative 2-oxo-4-hydroxy-4-carboxy-5-ureidoimidazoline decarboxylase	25
SPATA7	Spermatogenesis-associated protein 7	25
LAMA1	Laminin subunit alpha-1	25
TGM2	Protein-glutamine gamma-glutamyltransferase 2	25
SMARCA1	Probable global transcription activator SNF2L1	24
PCLO	Protein piccolo	24
PEX10	Peroxisome biogenesis factor 10	24
HIST1H1E	Histone H1.4	23
TRIM29	Tripartite motif-containing protein 29	23
MRPL13	39S ribosomal protein L13, mitochondrial	23
PRPF19	Pre-mRNA-processing factor 19	22
HIST1H1B	Histone H1.5	22
RPL30	60S ribosomal protein L30	21
RPL27	60S ribosomal protein L27	21
HNRNPH1	Heterogeneous nuclear ribonucleoprotein H	21
MRPL53	39S ribosomal protein L53, mitochondrial	20
ARHGEF17	Rho guanine nucleotide exchange factor 17	20
RBM15	Putative RNA-binding protein 15	20
